# Foliar fungal communities in agroecosystems depend on crop identity and neighboring vegetation

**DOI:** 10.3389/frmbi.2023.1216462

**Published:** 2023-07-13

**Authors:** B. K. Whitaker, R. W. Heiniger, C. V. Hawkes

**Affiliations:** ^1^USDA, Agricultural Research Service, National Center for Agricultural Utilization Research, Mycotoxin Prevention & Applied Microbiology Unit, Peoria, IL, United States; ^2^Department of Plant and Microbial Biology, North Carolina State University, Raleigh, NC, United States; ^3^Department of Crop and Soil Sciences, North Carolina State University, Raleigh, NC, United States

**Keywords:** wheat, corn, soy, switchgrass, mycobiome, microbiome, landscape, ecology

## Abstract

Agricultural intensification causes plant diversity loss and environmental homogenization, which may result in changes to plant-microbiome interactions mediating plant growth and stress tolerance. We hypothesized that foliar fungal microbiomes would depend on plant traits and environmental filters, constrained by neighboring vegetation expected to serve as a fungal source. Thus, we sampled foliar fungi from four crops (three annual and one perennial), four sites per crop, and three varieties per annual crop, across a 500-km expanse in North Carolina, USA and tested the role of host traits, environmental traits, and vegetative landcover on foliar fungal community structure. Crop species and site were major determinants of community structure, primarily due to differences in plant size and growing season. Site consistently explained 10× more variation in community structure than host variety across the annual crops. Finally, reduced natural vegetative cover surrounding farms was correlated with decreased fungal richness and more homogeneous microbiome assembly. Based on these results, we posit that foliar fungal assembly in crops results from host and environmental filters acting on inputs from the nearby vegetation. Future efforts at agricultural microbiome management must therefore consider landscape management and will require an improved understanding of how agricultural intensification alters microbial source pools.

## Introduction

In native ecosystems, assembly of the plant microbiome is generally driven by host diversity and environmental heterogeneity, which serve as both microbial propagule sources and selective filters (Balint et al., 2015; Giauque and Hawkes, 2016; Oono et al., 2017; Whitaker et al., 2018; Maciá‐Vicente and Popa, 2021). Although the same assembly processes should control crop microbiomes, it is not clear whether the dominant processes are niche- (biotic/host or abiotic/environmental filters) or dispersal-based. Intensive agricultural practices negatively impact overall microbial biodiversity (Rodrigues et al., 2013; Karlsson et al., 2017; Li et al., 2019), while increasing pathogen incidence and severity (Claflin et al., 2017; Ingwell et al., 2017). These microbial shifts likely reflect limited crop diversity and environmental homogenization that affect both local assembly filters and microbial propagule supply (Soldan et al., 2021).

In natural ecosystems, increased plant diversity means greater variation in plant traits and more potential plant-based niches for microbial taxa. In modern agriculture, plant diversity loss has been two-fold: fewer crops and more genetically similar varieties have reduced both inter- and intraspecific plant diversity. Selective breeding for agronomically desirable traits, including pathogen resistance, grain yields, and stand density, is associated with microbiome divergence between crops and wild relatives (Hassani et al., 2020). Similarly, intraspecific trait differences can act as selective biological filters to microbiome assembly (Wallace et al., 2018; Simonin et al., 2020; Wagner et al., 2020) with convergence more likely when varieties are ecologically similar (Ricks and Koide, 2019). Nevertheless, host effects on microbiome assembly appear to be weaker than environmental effects (Balint et al., 2015; Whitaker et al., 2018), suggesting that plant traits (whether inter- or intraspecific) must be considered in an environmental context.

Environmental filters in the form of abiotic conditions structure microbiomes across multiple spatial scales. For example, in natural ecosystems, water availability drives microbial survival across micro- (e.g. biofilms; Lennon et al., 2012) to macro-scales (e.g. climatic regions; Bahram et al., 2018). In agricultural systems, both micro- and macro-scale environmental homogenization associated with modern farming (e.g. tillage, field enlargement; Ramankutty et al., 2018) has likely altered the primary environmental filters acting on microbiomes. Moreover, consequent niche disparities between natural and agricultural systems raise the question of possible microbial exchanges between the two systems (Susi and Laine, 2021).

Microbial dispersal affects microbiome assembly *via* taxa supply across local (from soil or plant to plant) to regional scales (across sites; Fort et al., 2016). In croplands, microbial reservoirs may decline following plant diversity loss, environmental homogenization, and increased disturbance (Laforest-Lapointe et al., 2017; Whitaker et al., 2021). Regional source-sink dynamics have been reported from decades of macro-organismal research (Ricketts, 2004; Tscharntke et al., 2005; Stanton et al., 2018), but our understanding of non-pathogenic microbial exchanges between agronomic and natural ecosystems remains limited. Empirical evidence from the crop phyllosphere suggests local bacterial exchange among mixed vegetable plantings (Meyer et al., 2022). And, although challenging to measure directly, landscape-level correlations between agricultural and native plant microbiomes indicate dispersal limitation (Jiao et al., 2021) and stringent local filtering (Vaz Jauri et al., 2018). Thus, low-diversity agricultural ecosystems may be microbial sinks that depend on spillover from surrounding natural or semi-natural areas.

To address drivers of crop microbiome richness and structure from local to landscape scales, we focused on the foliar fungal community associated with four crops each grown at four sites across a 500-km region of North Carolina (NC), USA. We selected foliar fungi because previous work demonstrated their dependence on plant-to-plant networks rather than soils as sources (Lee and Hawkes, 2021; Whitaker et al., 2021). We sampled three annuals (corn, soy, and wheat; n=3 varieties per crop) and one perennial (switchgrass). For each site, we collected data on: plant performance traits that might act as biotic/host filters (Lee and Hawkes, 2021), climate and soil characteristics that are likely environmental filters (Giauque and Hawkes, 2016); and vegetative cover in the surrounding landscape as a metric of potential fungal sourcing, as seen in wild communities (Whitaker et al., 2021).

**Figure 1:**
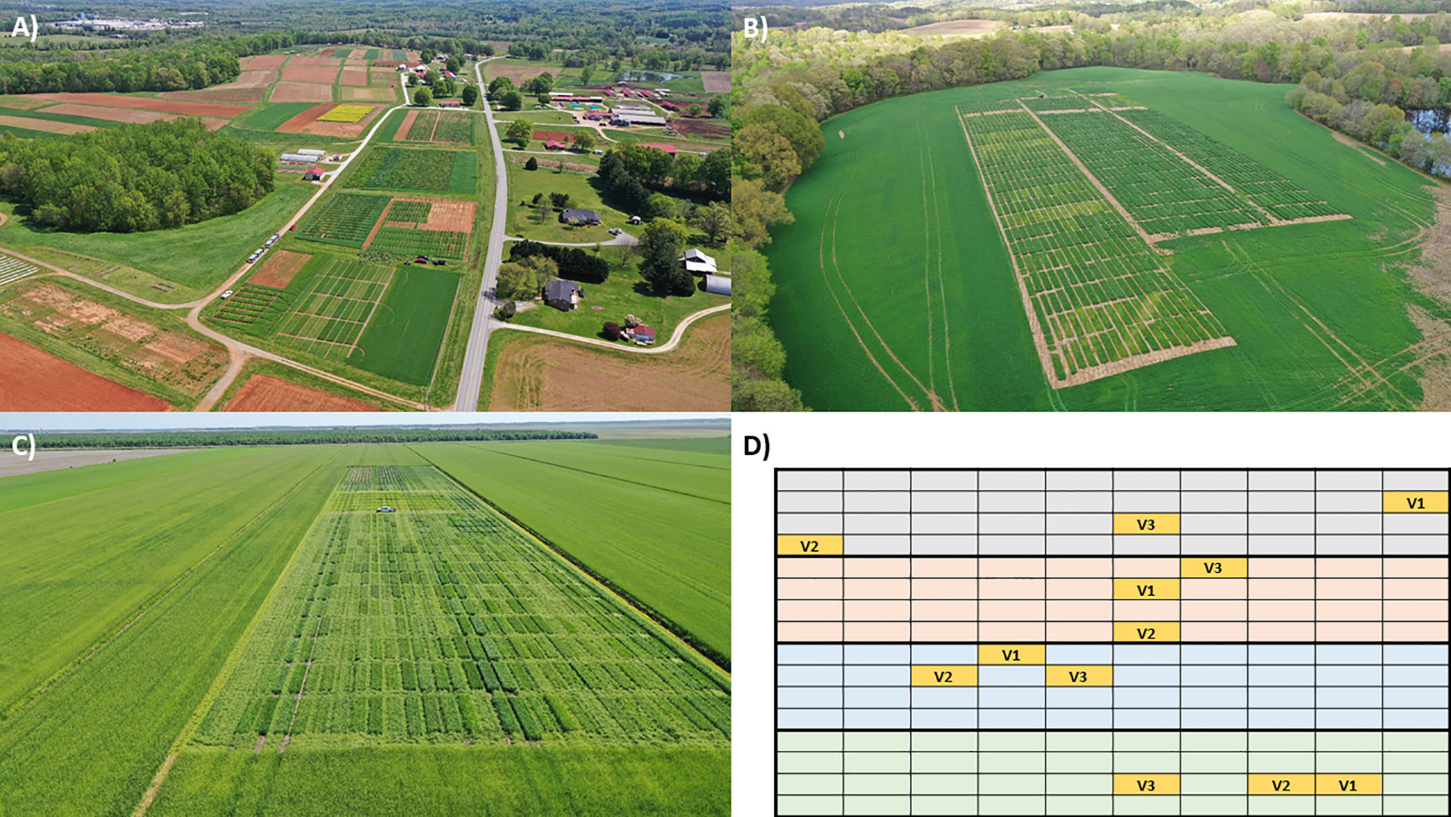
Examples of **(A–C)** three experimental sites in the North Carolina State University’s Official Variety Testing program and **(D)** a randomized block design where each block is represented by a different color and the plot locations for the three sampled varieties (V1, V2, V3) are indicated in yellow. In addition to site differences such as climate and soil type, each site is surrounded by landscapes with different amounts and types of land cover. Photos by Ryan Heiniger.

We tested three interrelated hypotheses. 1) We expected crop microbiome structure and richness to vary across host species and by the interaction between host variety and sites, assuming a combination of plant traits and environment would act as selective filters. 2) We expected site effects would better predict microbiome structure than variety effects based on the dominance of environmental filters in other studies (Wallace et al., 2018; Whitaker et al., 2018; Wagner et al., 2020), even though host varieties were selected to maximize variation in agronomic traits. 3) Lastly, we hypothesized that differences across sites and plots in crop microbiome structure, richness, and dissimilarity would reflect a combination of host filters (plant traits), environmental filters (climate and soil traits), and fungal propagule supply. Specifically, we predicted that the abundance of unmanaged, natural/semi-natural vegetative cover in the surrounding landscape (**Figures 1A–C**) would correlate with fungal community metrics based on the assumption that unmanaged vegetation acts as a fungal source to farms.

## Materials and methods

### Study sites and crop species

To represent a range of growing conditions, we sampled from four crops (with four sites per crop = 16 fields) across three biogeographic regions in NC: Mountains, Piedmont, and Coastal Plains (**Supplementary Figure S1**). We sampled three annuals: (*Glycine max* L. [hereafter soy], *Triticum aestivum* L. [soft red winter wheat, hereafter wheat], and *Zea mays* L. [hereafter corn]) from North Carolina State University’s Official Variety Testing (OVT) sites (https://officialvarietytesting.ces.ncsu.edu; **Supplementary Table S1**). We also sampled perennial *Panicum virgatum* L. (hereafter switchgrass) from monoculture stands (**Supplementary Table S1**). For each annual, three varieties were selected to maximize variation in yield, pathogen resistance, stress tolerance, and GMO traits (**Supplementary Table S2**). Variety testing was not possible for switchgrass (**Supplementary Table S2**), but previous studies detected little to no influence of genetic type on switchgrass leaf microbiomes (Whitaker et al., 2018; Hestrin et al., 2021).

### Experimental design and sampling

For annual crops, we sampled varieties from the OVT sites where plots were organized in a complete randomized block design (3 varieties × 4 blocks per site; **Figure 1D**). Each plot measured 1.2m × 7.6m and planting density varied by crop: 2 rows (corn), 4 rows (soy), or 7 rows (wheat) with 76.2cm, 38.1cm, or 17.8cm row spacing, respectively. Plot sizes were the same for switchgrass – except at WBFL (plot sizes 2.4m × 4.9m) – with no cultivar replication, 3-4 blocks per site, and 4 or 3 plots per block (respectively). For complete details of switchgrass sampling, see Supplementary Methods.

We sampled all crops in 2019 during either flowering or grain/pod filling: Apr 30-May 10 (wheat: Feekes 10.5-10.5.1), Jul 24-Aug 2 (corn: Abendroth R3-R4; soy: Fehr and Caviness R1-R3), or Aug 7-19 (switchgrass: flowering). Specific leaves were targeted based on crop identity and growth form: ear leaf (corn), flag leaf (switchgrass, wheat), or leaflets from the 3^rd^ and 4^th^ highest fully expanded leaves (soy). One leaf, or leaflet set, was collected from four randomly selected plants per plot, combined, stored on dry ice, and transferred to a -80°C freezer on the same day.

### Plot and site data collection

Measured variables represented potential microbial biotic and abiotic filters. For each plot, we recorded latitude and longitude, and measured plant height, leaf area index (LAI), and soil moisture from the same plants sampled for leaf tissue. Plant height and LAI were proxies for plant performance because they predicted vegetative yield in switchgrass (Aspinwall et al., 2017) and because height was strongly correlated with grain/pod yields (Adj. R^2 ^= 0.92). For additional details, see the Supplementary Methods.

For each site, we obtained historical climate (1981-2010) and 2019 weather data, including: mean annual high and low temperatures (MHT, MLT) and precipitation (MAP), and high and low temperatures and accumulated precipitation for 90 days preceding sampling (HT_90day, LT_90day, and Precip_90day; Prism Climate Group, 2021). As broad indicators of soil properties, we measured soil pH and humic organic matter (%HM) from the annual crops at sampling; for switchgrass, these data were obtained from soils collected in 2018 (Supplementary Methods). Location, climate, and soil type/texture are provided for each site (**Supplementary Table S3**).

To account for potential microbial source-sink exchange, we computed the percentage of unmanaged natural/semi-natural vegetation and active cropland within 1- and 10-km radii of each site and plot using the USDA-NASS Cropland data layer (dates: 01/01/2019-12/31/2019, resolution: 30m; USDA-NASS, 2019) processed in Google Earth Engine (Gorelick et al., 2017; Supplementary Methods, **Figures S2**, **S3**). These spatial scales represent nearby farm edges and more distant vegetation, respectively, given typical NC farm sizes of ~0.5km^2^ (USDA-NASS and NCDA-CS, 2019).

### Illumina sample preparation

Leaves from each plot were randomly sub-sampled by cutting tissue on frozen cutting boards into 0.25cm^2^ fragments. DNA was extracted using the Synergy 2.0 Plant DNA Kit (OPS Diagnostics LLC, Lebanon, NJ). We pooled one DNA blank per kit as a negative control prior to PCR amplification. For Illumina library preparation, we performed two-stage PCR with modified ITS1F and ITS2 primers (Smith and Peay, 2014) and a custom-designed peptide nucleic acid (switchgrass samples only). For details, see Supplementary Methods, **Figures S9**, **S10**, and **Table S12**. Raw sequencing data are available in NCBI SRA BioProject PRJNA845782 (SRR19545648 – SRR19545890).

### Bioinformatics

All sequence data were processed using DADA2 (v.1.14.1; Callahan et al., 2016) to determine amplicon sequence variants (ASVs) with default parameters except filtering (maxEE=2,4). Plant sequences were removed using a custom database, followed by LULU curation (v.0.1.0; Frøslev et al., 2017). Taxonomy was determined using the RDP naïve Bayesian classifier (Wang et al., 2007) and Warcup fungal ITS database (v2; Deshpande et al., 2016), with substitutions from the UNITE fungal ITS database (Kõljalg et al., 2013) for confidence scores <70%. For details, see Supplementary Methods.

### Statistical analyses

All bioinformatics and statistical analysis scripts are archived on Zenodo (Whitaker, 2023). All analyses were run in R (v.4.2.0; R Core Team, 2022), with substantial use of phyloseq (McMurdie and Holmes, 2014), vegan (v.2.6-2; Oksanen et al., 2022), and DESeq2 (v.1.32.0; Love et al., 2014). All data figures were created using ggplot2 (v.3.3.5; Wickham, 2016). The dependent variable in all community structure analyses was a Euclidean distance matrix (McMurdie and Holmes, 2014) from variance-stabilized ASVs (DESeq2), to account for compositionality and overdispersion (Gloor et al., 2017).

To address our first hypothesis that fungal community structure and richness varied across crop species, variety, and site, we used a series of linear models *via* residual randomization permutation procedures (RRPP, v.1.1.1; 1,000 permutations; Collyer and Adams, 2018). RRPP allows for Type III sum of squares and complex mixed model designs *via* explicit selection of the denominator for pseudo-*F*-ratio calculations. First, we tested crop species (fixed), site nested in crop (fixed), and block nested in the crop-by-site interaction (random). Second, we tested each annual crop separately with site, variety, and their interaction as fixed effects, and included both the block within site and block within site-by-variety interaction, as non-testable, random error terms. Third, for switchgrass, no variety effect was included; thus, block nested within site could be tested and was also used as a random error term.

To address our second hypothesis that site would better predict microbiome structure than host variety, we identified their relative contributions to community structure for each crop separately, along with nested block effects, using variance partitioning in db-RDA (vegan::varpart, capscale). Significance testing was performed on each variable using conditional and partial conditional permutation tests (n=999).

To test our third hypothesis (and delve further into unexplained variation identified in the db-RDA), we investigated the selective filters driving microbiome structure, richness, and dissimilarity. We analyzed site-level community structure with partial canonical correspondence analysis (CCA; vegan::cca) and site-level richness and dissimilarity with best subsets ordinary least squares linear regression. In each analysis, we initially included the following site-level predictors: climate (MHT, MLT, MAP), elevation, recent weather (HT_90day, LT_90day, Precip_90day), landcover (percent unmanaged vegetation and percent cropland at 1- and 10-km scales), soil properties (pH, % HM, soil moisture), and crop productivity traits (average LAI and height). The WHT-PASVT site was missing soil data, thus we performed two versions of the site-level analyses: 1) WHT-PASVT excluded but soil characteristics included and 2) WHT-PASVT included but soil characteristics excluded. Results were qualitatively similar (reported in **Supplementary Tables S8**, **S9**) and no soil properties were significant. Therefore, we chose to present the analysis with all n=16 sites but no soil characteristics.

For the CCA, latitude and longitude were the conditioning matrix, while the response matrix was the Euclidean distance matrix of the variance-stabilized ASVs pooled by site. Independent variables were successively dropped by largest variance inflation factor (VIF) until all VIFs<2, leaving MAP, Precip_90day, LT_90day, elevation, percent unmanaged vegetation within 1-km, soil moisture, and mean LAI. Permutation tests were used to assess the significance of variables and axes (n=999; vegan::envfit, anova.cca).

Average within-site fungal community richness and dissimilarity were examined with regression as a function of the same explanatory variables listed above. Within-site dissimilarity was calculated as the average of all between-plot Euclidean distances (12 plots per site, 66 total comparisons per site). For VIFs<2, the model included: MAP, Precip_90day, LT_90day, percent unmanaged vegetation at the 1-km scale, soil moisture, and mean LAI. The best models were determined using Akaike Information Criteria (AIC), Schwarz Bayesian Criteria (SBC), and Adjusted R^2^ (olsrr::ols_step_best_subset, v. 0.5.3; Hebbali, 2020).

Lastly, any significant site-level effects that were also measured at the plot scale were reanalyzed for plot-level richness and dissimilarity using mixed effects regressions (nlme::lme, v.3.1.161; Pinheiro and Bates, 2023). This allowed us to examine the effect of crop on these relationships. Plot dissimilarity was calculated as the average Euclidean distance between the target plot and every other plot at the same site (11 comparisons). Crop species was tested either as a random intercept or as both random intercept and slope. AIC was used to determine the best model structure.

## Results

There were 595 total ASVs. Most ASVs were identified as Ascomycota (64.0%) or Basidiomycota (32.4%). The top ten most abundant ASVs accounted for 48.2% of sequencing reads; six belonged to the Mycosphaerellaceae (**Supplementary Figure S4**). Only 5 ASVs (<1%) were common across all sites (*Davidiella* sp., *Alternaria* sp., and three *Phoma* spp.), while 169 ASVs (28.4%) were unique to one site. The four crop species shared 57 ASVs (9.6%%; 1.8-fold more Asco- than Basidiomycota), whereas 323 ASVs (54.3%) were unique to one crop species (**Supplementary Figure S5**). Switchgrass had the largest portion of unique ASVs (24.0%), followed by corn (16.1%), wheat (10.3%), and soy (3.9%). There were more unique Asco- than Basidiomycota in corn, wheat, and switchgrass, but there were more unique Basidiomycota in soy.

**Figure 2:**
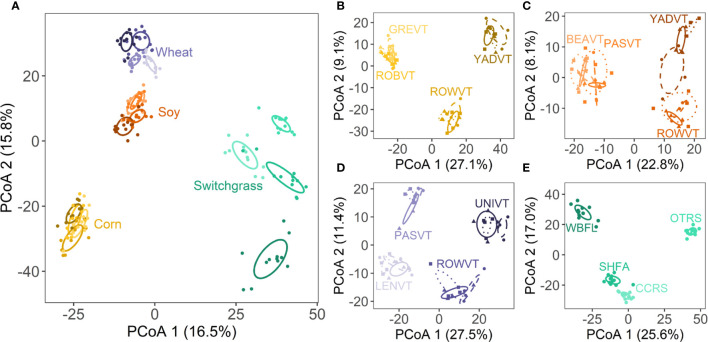
Ordinations depict differences among **(A)** crop species and sites; the site by variety interaction for **(B)** corn, **(C)** soy, and **(D)** wheat; and **(E)** sites for switchgrass. Each point represents a single plot. Ellipses represent 1 SE around the centroid for each treatment group. Specific color shades represent different sites as labelled in panels **(B–E)**. For **(B–D)** specific line types and point shapes represent the three different varieties per annual crop. For full site names, see **Supplementary Table S3**.

*Hypothesis 1* ***–*** Fungal community structure (**Figure 2A**) and richness (**Supplementary Figure S6**) differed among crops (both *P*<0.001) and site nested in crop (both *P*<0.001). Community structure also varied spatially among blocks nested in site (*P*<0.001), whereas richness did not (*P*=0.825; **Supplementary Table S4**). When corn, soy, and wheat were analyzed separately, there was an interaction between site and variety on fungal community structure (all *P*<0.001; **Supplementary Table S5**; **Figures 2B–D**). For switchgrass, fungal community structure varied across sites (*P*<0.001) and blocks nested within site (*P*<0.001; **Supplementary Table S5**; **Figure 2E**). Site, variety, and block effects on fungal richness followed a similar pattern to community structure for all crop species (**Supplementary Table S6**).

*Hypothesis 2* ***–*** Based on variance partitioning, site explained more variation (11-13%) in community structure than host variety (0-2%) in the annual crops (**Supplementary Table S7**; *P*<0.001). Block effects, a metric of within site-spatial variation, were minimal (<3%) and only significant for corn (*P=*0.047) and switchgrass (*P*<0.001). However, the variation explained by the shared portion between site and block was high in all four crops (20-34%). Most fungal community structure variation was unexplained (46-70%) by these experimental factors (**Supplementary Table S7**).

**Figure 3:**
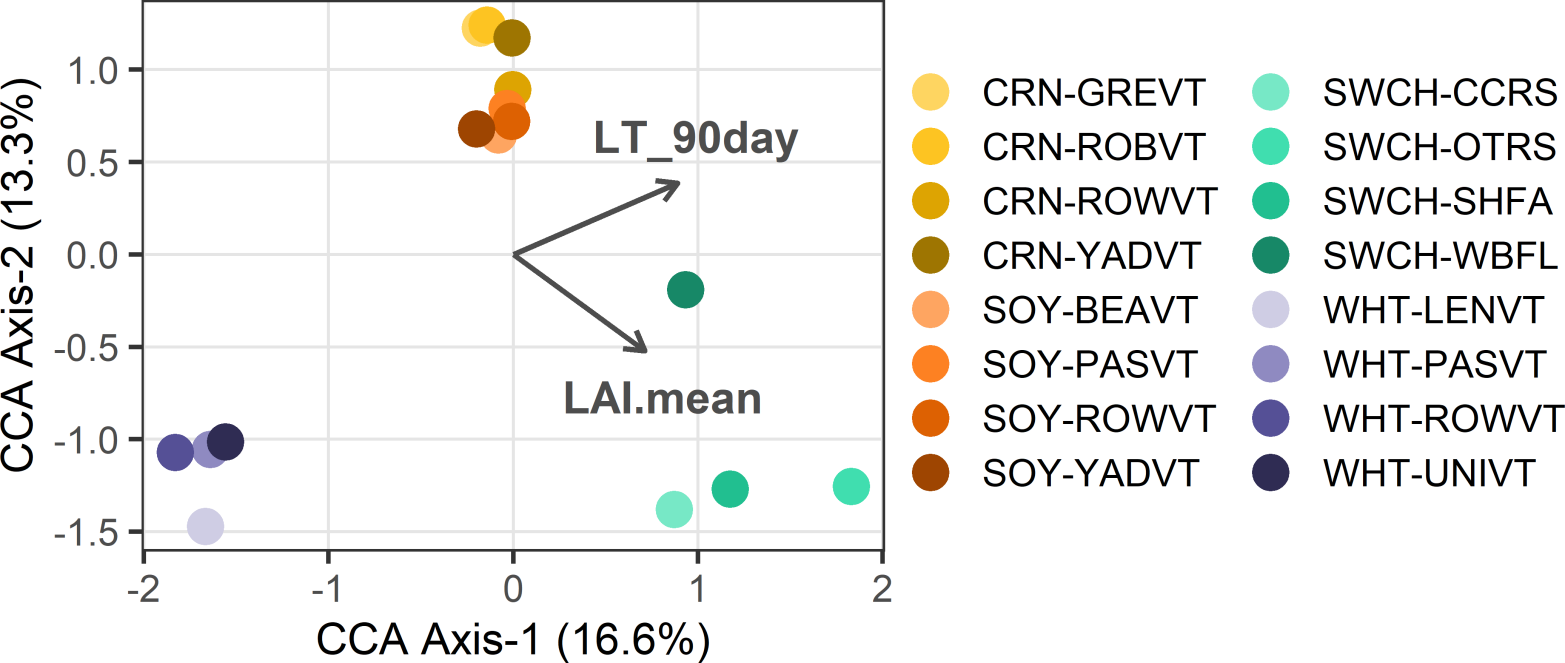
Partial CCA results are shown where each point represents the crop foliar fungal community at a single site. Vectors depict significant continuous traits driving community structure: ‘plantHt’=mean plant height, ‘LAI.mean’=mean LAI, ‘Perc_Veg_10km’=% Unmanaged Vegetation at 10-km scale, and ‘LT_90day’=low temperature in 90 days preceding sampling. Colors represent crops and color shades indicate sites.

*Hypothesis 3* ***–*** To parse the role of biotic and abiotic environmental factors in fungal community structure across sites, we used partial CCA controlling for spatial location (**Supplementary Table S8**; **Figure 3**). Only CCA Axis 1 was significant (*P=*0.041). Fungi in perennial switchgrass and the three annual crops separated along CCA Axis 1 based on plant LAI (*P=*0.001), which was 1.8× higher in switchgrass relative to the annual crops. Fungi in spring-maturing wheat also separated from the summer-maturing crops based on 90-day low temperature (*P=*0.001).

**Figure 4:**
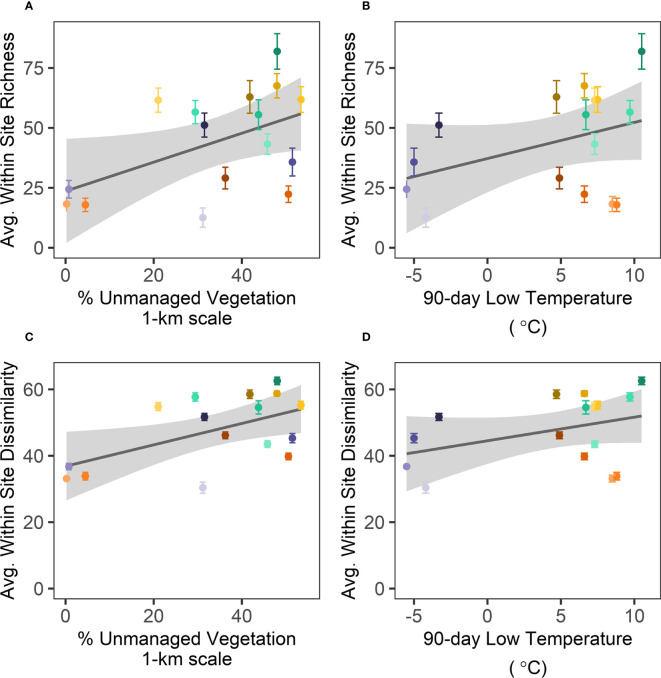
Across sites, average fungal richness **(A, B)** and dissimilarity **(C, D)** are positively related to **(A, C)** unmanaged vegetation at the 1-km scale and **(B, D)** with low temperatures in the 90 days preceding sampling. Each point represents the average (± 95% CI) observed richness or community dissimilarity between plots within a site and are color coded according to crop species and site as in **Figures 2**, **3**. Gray bands show ± 1 SE for the linear regressions.

Best subsets regression analysis revealed that unmanaged vegetation at the 1-km scale and low temperatures in the 90 days preceding sampling best predicted both average site-level fungal richness (Adj. R^2 ^= 0.29; **Supplementary Table S9**; **Figures 4A, B**) and community dissimilarity (Adj. R^2 ^= 0.33; **Supplementary Table S9**; **Figures 4C, D**). Other variables from the top five models included measures of water availability (MAP, Precip_90day, soil moisture) or LAI, but these did not improve model fit.

The plot-level analysis recapitulated site-level patterns between 1-km unmanaged vegetation with fungal community richness and dissimilarity (both P < 0.001; **Supplementary Table S10**; **Figure S7**). The best fit for plot-level fungal richness included crop as random intercepts, such that all crops have the same slope with 1-km unmanaged vegetation but different means. For plot-level fungal dissimilarity, AIC could not distinguish the random intercepts model from the random intercepts and slopes model (**Supplementary Table S10**), such that the effect of 1-km unmanaged vegetation on dissimilarity might be crop-specific.

## Discussion

Crop species were colonized by different fungal microbiota, reflecting both seasonal environments and size-based host traits that can act as filters on microbial community assembly. As expected, host variety explained substantially less variation than site in foliar fungal community composition. Most differences observed across sites were primarily attributed to seasonal turnover and unmanaged vegetation found within 1 km of the farms. Within sites, crop fungi were heterogeneous, with the degree of heterogeneity also reflecting seasonal turnover and nearby unmanaged vegetation. Thus, a working model is that neighboring vegetation serves as a potential fungal propagule source to crop leaves, where successful colonization is dictated by plant trait and environmental filters that together control both microbial diversity and composition. These results emphasize the importance of landscape context and the need to better understand plant traits and seasonal turnover as potential filters for specific microbial niches.

Host filtering is a common constraint on microbiome assembly. We found strong local biotic filters, as evidenced by variation in fungal community structure across crop species and, to a lesser extent, host varieties. One crop trait, LAI, was an important correlate of fungal community structure. Vegetative growth traits can influence niche space for colonizing fungi, for example by increasing available habitat (Meyer et al., 2022) or changing micro-climate (Vidal et al., 2017). Across annual crops, varieties showed minimal differences in foliar fungi, which could reflect our variety selection. Larger host variety effects on fungal communities might occur if there were greater contrasts in relevant traits, such as fungal pathogen resistance (Wagner et al., 2020) or phenology (Sutherland et al., 2022).

Environmental filters that affected foliar fungal community structure, richness, and dissimilarity were limited to recent low temperatures, reflecting the seasonal difference between spring and summer sampling for wheat versus all other crops. This likely reflects both seasonal variation in natural vegetation source pools, with fewer and different types of green plants in the surrounding landscape in the spring, as well as differences in fungal environmental tolerance. In natural ecosystems, climate (Giauque and Hawkes, 2016) and soil characteristics (Lee and Hawkes, 2021), are often dominant environmental drivers of the plant fungal microbiome. In agricultural ecosystems, these influences may be mediated by source population availability or may be inconsequential compared to the disturbance associated with intensive agricultural management (Karlsson et al., 2017; Ricono et al., 2022). Here, with only one spring crop tested, our ability to interpret this result is limited – as it might be caused by wheat-specific traits not measured here. To fully separate fungal propagule supply from other biotic and abiotic filters will require studying microbial diversity across a greater number of spring and summer maturing crops.

Unmanaged vegetation in the 1-km surrounding each site, which ranged from 1-53% cover (**Supplementary Figure S3**), serves as a potential source of foliar fungi to crop leaves and likewise diversifies potential outcomes in crop microbiome community assembly. Specifically, decreased cover of nearby unmanaged natural/semi-natural vegetation was correlated with fewer fungal taxa and more similar microbiomes across plots within the same field site explaining 21% and 27% of variation in the data, respectively. This was likely due to a loss of fungal species diversity in surrounding source pools. The effect of nearby vegetation on fungal richness was consistent across crops, but the effect on fungal dissimilarity may be crop-specific and will require additional studies to fully elucidate. Along these lines, Ricono et al. (2022) found that hedgerow density had a neutral to negative effect on wheat root endophyte richness, suggesting that these may not be simple source-sink relationships.

Control of microbial pathogens represents an important focus of agricultural management and could be a concern in agriculture-native ecosystem exchanges. For example, genetic diversity of surrounding plant vegetation caused more frequent or severe disease in certain crops (Claflin et al., 2017; Ingwell et al., 2017). Here, all foliar tissue used for microbiome analysis was asymptomatic and only a few sites showed foliar disease symptoms (e.g. LENVT-Wheat, YADVT-Corn). We attempted to assess the pathotrophic fungal community using FUNGuild (Nguyen et al., 2016). Unfortunately, only 35% of ASVs could be confidently assigned to a single functional group (patho-, sapro, or symbio-troph); of these, 48% were pathotrophs (Supplementary Methods). The paucity of assignments makes interpretation difficult, but pathotrophic ASVs tracked the same variables as overall foliar fungal richness and dissimilarity (**Supplementary Figure S8**). This suggests that asymptomatic pathotrophs are proportionally abundant in the local/regional species pool and/or that there is little discrimination in fungal dispersal or selective filtering onto crops.

Despite large differences in foliar fungi among the four crops, ~10% of ASVs were shared, which likely reflects a set of generalist fungi with widespread dispersal. Five ASVs appeared at all sites: three *Phoma* spp., a *Davidiella* sp., and an *Alternaria* sp. All three genera are frequently identified as leaf endophytes (Arnold, 2007; Busby et al., 2016) and the latter two can be abundant in the corn phyllosphere (Singh and Goodwin, 2022). Alternatively, some fungi shared across different crops may not be generalists, but instead may respond to plant traits that represent similar niche spaces. For example, corn and switchgrass are both large C4 grasses and shared the most taxa (151 ASVs), despite a minimum distance of 92km between sites. To truly track fungal strains across crops and landscapes will require finer genetic resolution (Wagner, 2021).

Further investigations of specific mechanisms driving foliar fungal sourcing in agricultural systems are warranted. For example, we did not identify fungi from native vegetation or directly consider natural vegetation characteristics beyond cover that might control fungal propagule supply, such as native host diversity, vertical structuring, edge effects, and fragmentation (Martin et al., 2019; Willing et al., 2021). Similarly, our survey of environmental, soil, and plant traits was not exhaustive, and other factors might be more relevant (Lee and Hawkes, 2021). Additionally, crop and site sampling were partially confounded with season and crop phenology. Previous work suggests that soils are not a substantive source of fungi for leaves (Lee and Hawkes, 2021; Whitaker et al., 2021), so we did not test this possibility. However, other studies have found that soils are an important reservoir of leaf bacteria (Grady et al., 2019) and thus the degree to which soils act as a source of microbiota may vary across different domains or functional groups.

In conclusion, crop species harbor distinct fungal communities selected from their environments. The loss of both crop diversity and natural or semi-natural habitats associated with agricultural intensification has ripple effects on multiple trophic groups above- and belowground (Robinson and Sutherland, 2002; Geiger et al., 2010). Here, we add to this list the potential supply of foliar fungi, where recent work has shown that microbial signatures can be a more important crop yield predictor than traditional measures such as physical or chemical soil properties (Asad et al., 2022). Additional follow-up studies will need to examine the relative relationships between foliar fungal symbionts’ function, landscape cover, and crop performance. Regardless, future efforts to manage foliar fungal symbionts for improved crop health in agricultural ecosystems should consider exchanges with natural ecosystems.

## Data availability statement

The datasets presented in this study can be found in online repositories. The names of the repository/repositories and accession number(s) can be found below: Raw sequencing data can be found on NCBI BioProject PRJNA845782 (https:://www.ncbi.nlm.nih.gov). Other data and R scripts can be found on Zenodo (https://zenodo.org/record/7888432#.ZFK9FnbMIuU,10.5281%2Fzenodo.788432).

## Author contributions

All authors conceived of the ideas and designed the experiments. Fieldwork and labwork were carried out by BW and RH. BW and CH analyzed the data. BW and CH wrote the manuscript and RH edited the manuscript. All authors contributed to the article and approved the submitted version.
