# Supplementary material for: Foliar fungal communities in agroecosystems depend on crop identity and neighboring vegetation
Source: Front Microbiomes. 2023 Jul 13;2:1216462. doi: 10.3389/frmbi.2023.1216462 (PMC12993485; doi:10.3389/frmbi.2023.1216462)
Supplement: Supplementary file 1 [file DataSheet_1.pdf]

ELECTRONIC SUPPLEMENTARY MATERIALS

Foliar fungal communities depend on crop identity and neighboring vegetation in agroecosystems

Whitaker, B.K, Heiniger, R., Hawkes C.V.

Author for correspondence: Christine V. Hawkes; Email: chawkes@ncsu.edu

Contents

	Description	Page
Figures		
Figure S1	Map of sites distributed across North Carolina	2
Figure S2	Examples of USDA NASS Cropland Data Layer and correlations	3
Figure S3	Landcover distributions across species, sites, and scales	4
Figure S4	Relative abundance of top ten most abundant ASVs	5
Figure S5	Venn Diagram of shared and unique taxa across crops	6
Figure S6	Observed fungal ASV richness for crop species and sites	7
Figure S7	Plot-level response to environment traits	8
Figure S8	Pathotroph community response to environment and plant traits	9
Figure S9	PNA design– Alignment of switchgrass ITS variants	10
Figure S10	PNA results– Proportion of plant reads in trial sequencing run	11
Figure S11	Plant eukaryotic amplification and sequencing depth across crops	12
Figure S12	Rarefaction curves for all samples	13
Figure S13	Accumulation curves for each crop segregated by site	14
Tables		
Table S1	Crop rotation information	15
Table S2	Crop variety information	16-17
Table S3	Site information	18-19
Table S4	Results of PERMANOVAs for fungal dissimilarity and richness	20
Table S5	Results of PERMANOVAs for fungal dissimilarity of each crop	21
Table S6	Results of PERMANOVAs for fungal richness of each crop	22
Table S7	Results of variance partitioning (redundancy) analysis	23
Table S8	Results of partial canonical correspondence analysis	24-25
Table S9	Results of OLS regression analyses for average site fungal richness & dissimilarity	26
Table S10	Results of mixed effects regressions for plot richness & dissimilarity	27
Table S11	USDA-NASS crop data layers	28-32
Table S12	Results of kmers tested for switchgrass specificity in PNA development	33
Methods		
	Switchgrass sampling design across sites	34
	Details on plant, soil, and vegetative cover measurements	34-35
	Illumina library prep and bioinformatics	35-37
	Switchgrass PNA design and testing	37-38
	FUNGuild assignment of Pathotroph community	38
	References	39-40

Figure S1 – Map of all sites across the state of North Carolina. Top left scale bar is 50 km. For full site names and descriptions see Table S3. The distance between sites ranged from 0.13 to 500 km and the elevation change across sites was 340 m.

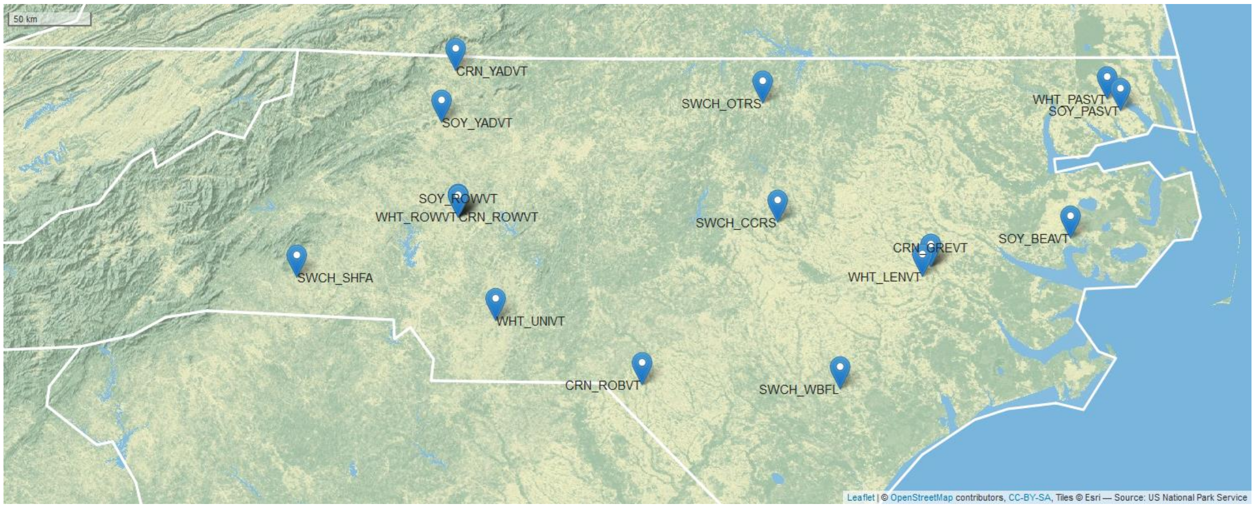

Figure S2 – Examples of USDA NASS Cropland Data Layer (CDL) imported into Google Earth Engine. The corn site GREVT is shown at the A-D) 1-km scale and E) 10-km scale. A) CDL masked to have colors represent various cropland types and gray represent all other landcover types, B) CDL masked to have dark-gray colors as cropland, C) CDL masked to have white and other light gray represent unmanaged vegetation, D) CDL masked to have light gray represent non-cropland/non-vegetation, and E) CDL masked to have colors represent various cropland types and gray represent all other landcover types. F) Strength and direction of the correlations between the four different site-level landscape metrics tested in the manuscript, across all sites.

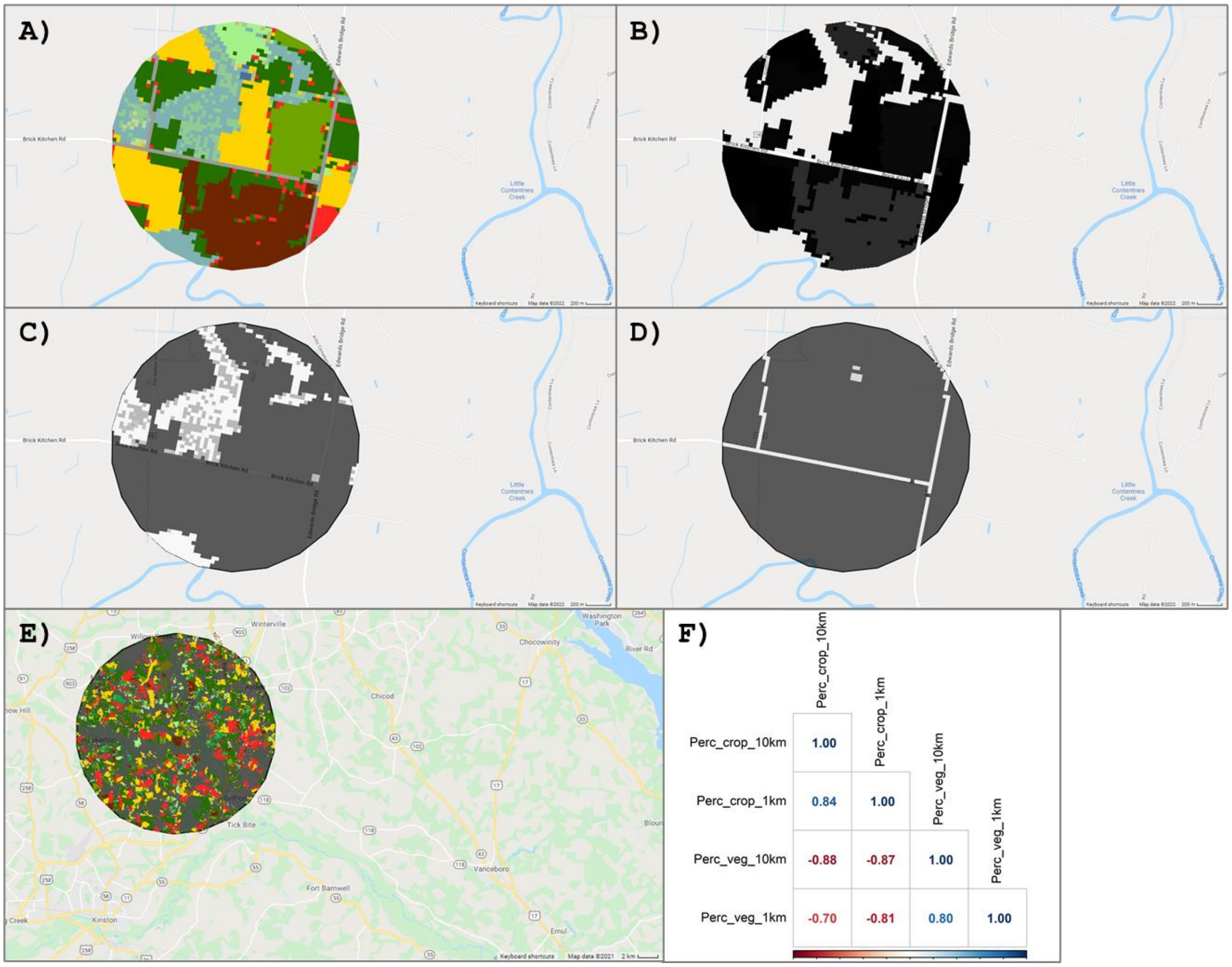

Figure S3 – Landcover distributions varied across species, sites, and spatial scales. Each point represents the percent of A, B) unmanaged vegetation or C, D) cropland for a single site, colored by species. These are measured at two scales, A, C) 10 km and B, D) 1 km. Violin plots show the distribution and range of land cover for each crop. . CRN = corn, SOY = soy, SWCH = switchgrass, WHT = wheat.

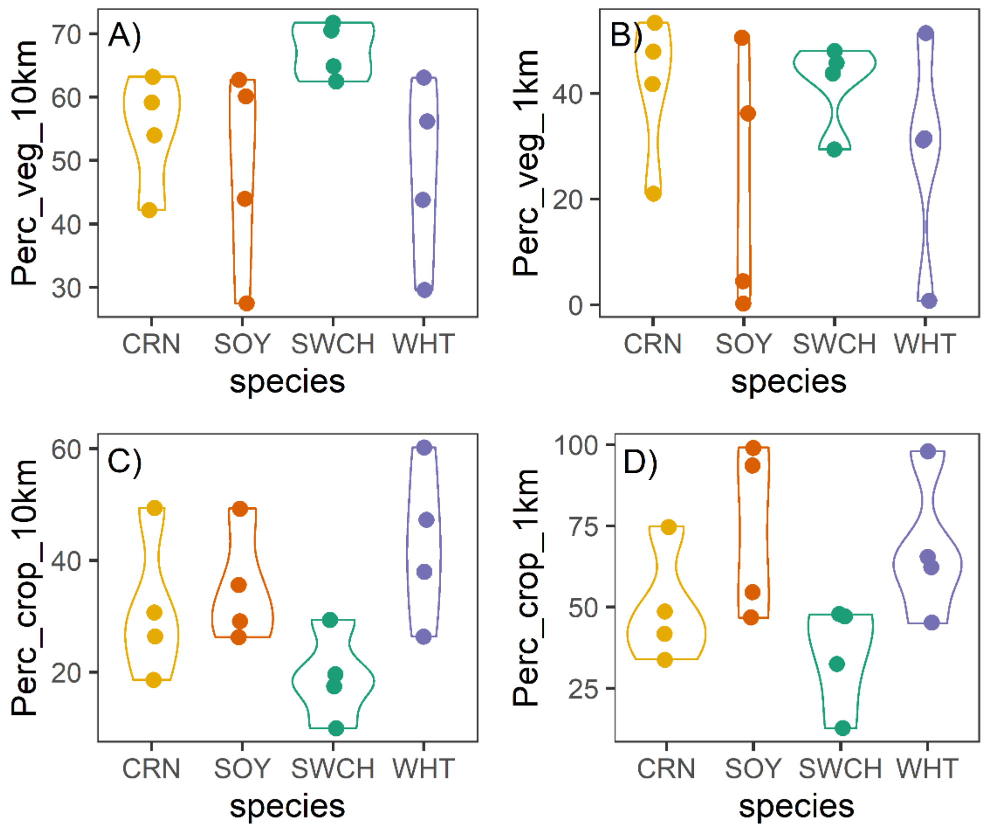

Figure S4 – Relative abundance of top ten most abundant ASVs by read count, depicted across crops, with all other taxa grouped into "Other". CRN = corn, SOY = soy, SWCH = switchgrass, WHT = wheat.

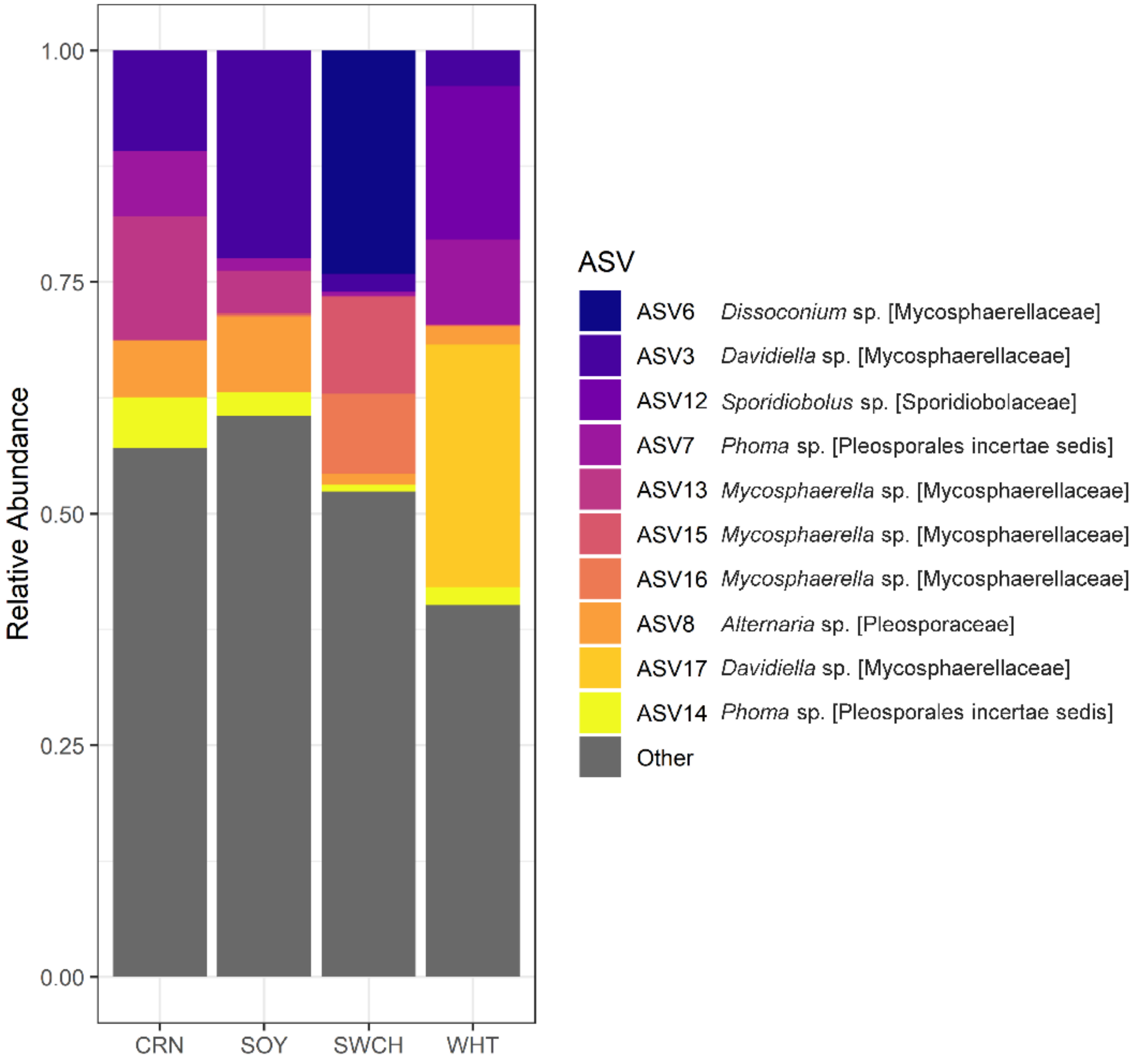

Figure S5 – Venn diagram of shared and unique ASVs across crops. Lighter to darker blue shades indicate increasing ASV counts in the set. CRN = corn, SOY = soy, SWCH = switchgrass, WHT = wheat.

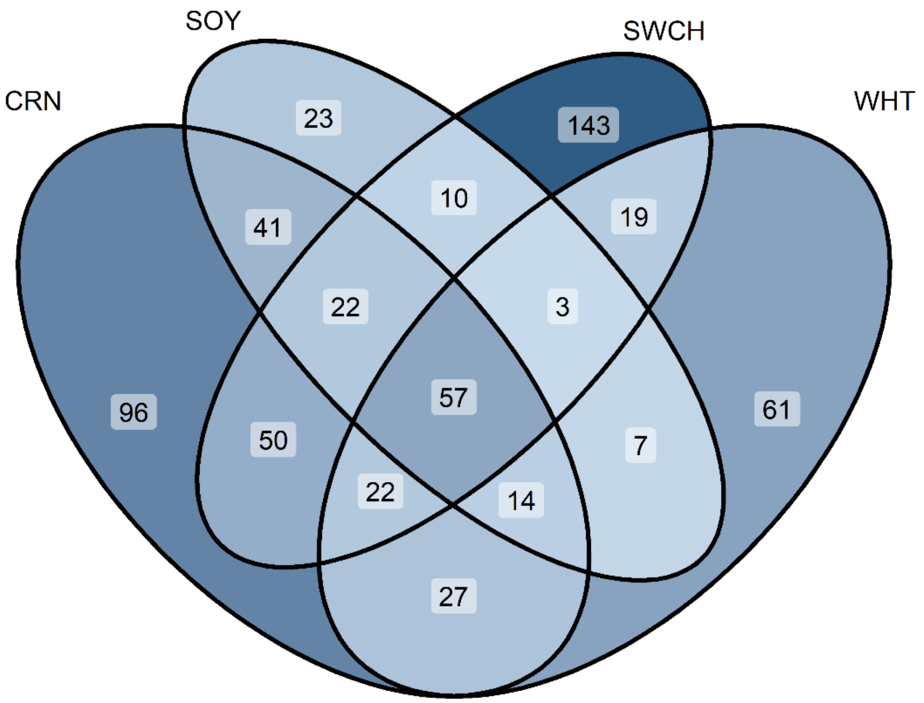

Figure S6 – Observed fungal ASV richness for each crop species at each site. Each point represents a single plot. Yellow = corn [CRN], orange = soy [SOY], green = switchgrass [SWCH], purple = wheat [WHT]. Specific color shadings represent different sites.

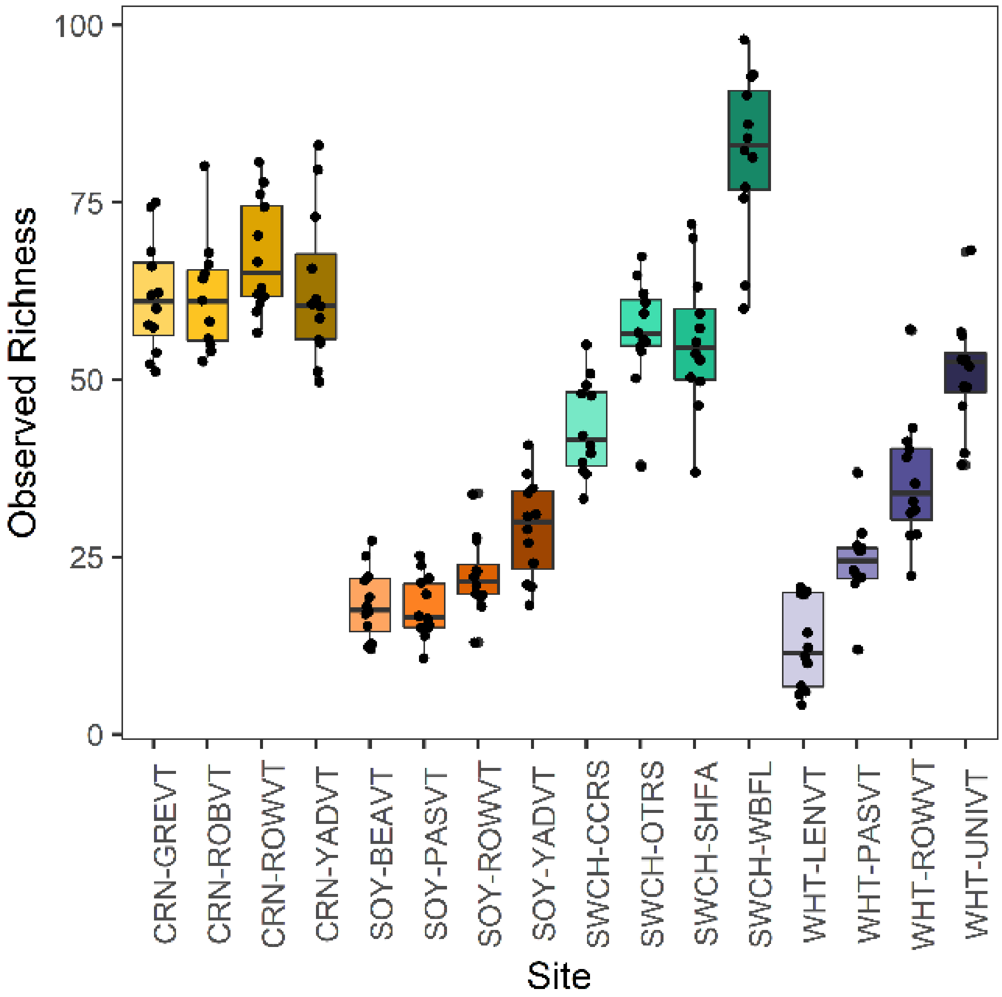

Figure S7 – Across plots, observed fungal richness (A) and dissimilarity (B) are positively related to unmanaged vegetation at the 1-km scale. Each point represents a single plot and are color coded according to crop species. Regressions depict model-based predictions for a random intercepts model and are also color coded according to crop species. Yellow = corn [CRN], orange = soy [SOY], green = switchgrass [SWCH], purple = wheat [WHT].

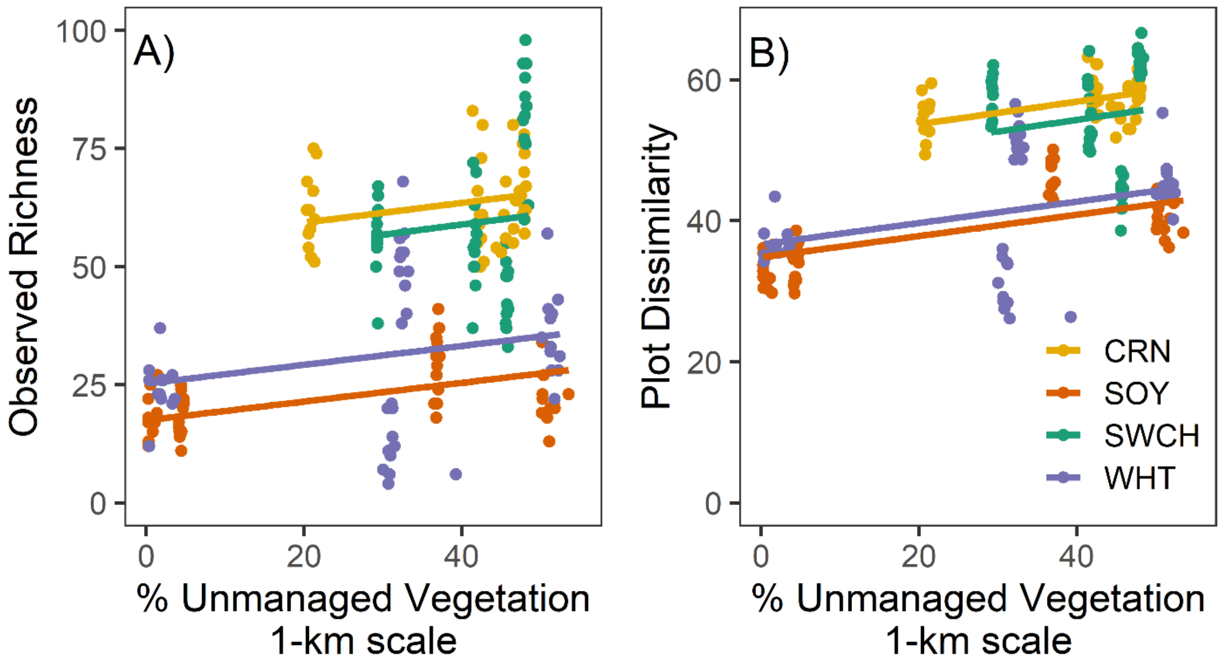

Figure S8 – The average (A, B, C) and standard error (D, E, F) proportion of pathotrophs across plots within sites are positively related to (A, D) unmanaged vegetation at the 1-km scale, (B, E) low temperatures in the 90 days preceding sampling, and (C, F) average Leaf-Area Index. Each point represents the average or standard error proportion of pathotrophs between plots within a site. Yellow = corn [CRN], orange = soy [SOY], green = switchgrass [SWCH], purple = wheat [WHT]. Specific color shadings represent different sites. Gray bands show ± 1 SE for the linear regressions.

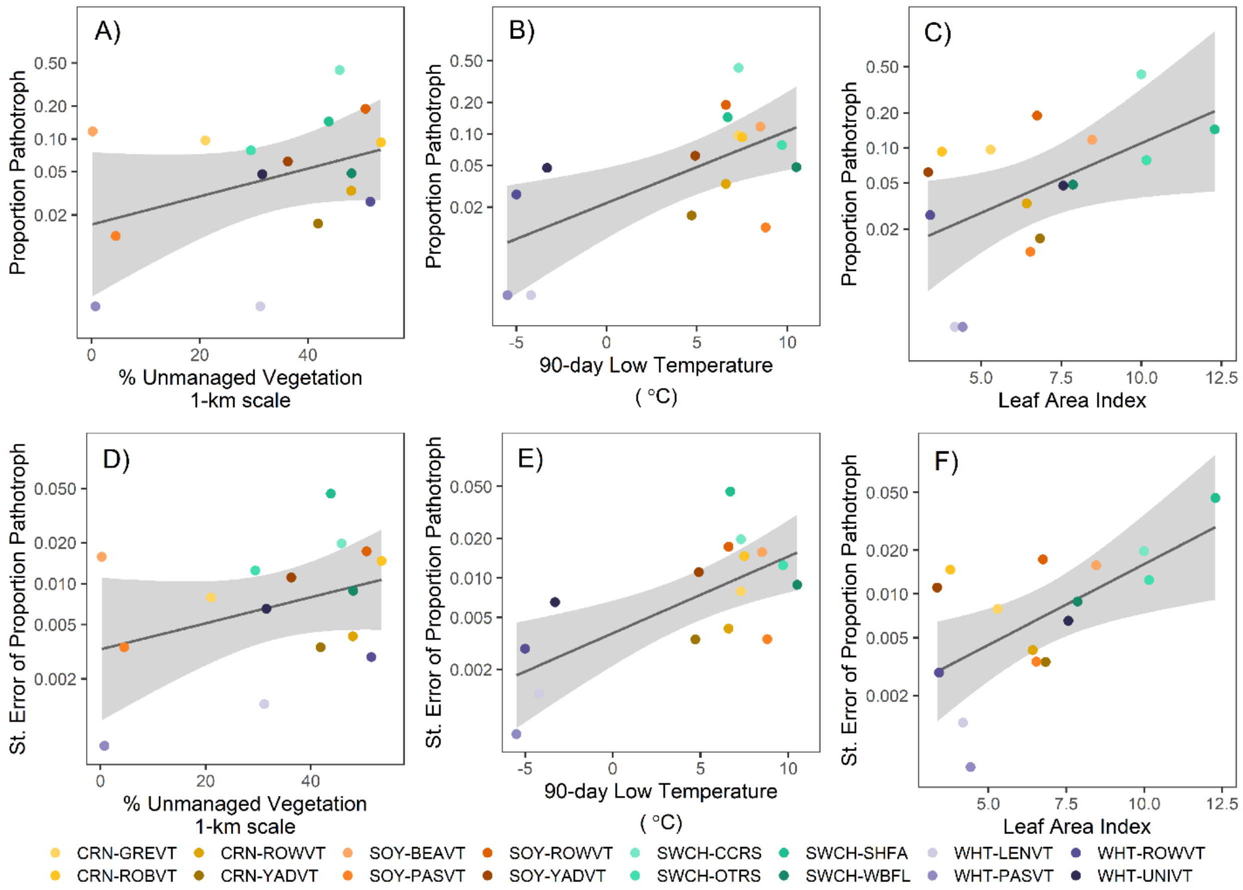

Figure S9 – Sequence alignment of switchgrass ITS variants derived from comparative genomics approach. Screenshot taken from Geneious showing MAFTT alignment of both intra- and intergenomic ITS sequences across four switchgrass genomes. Green and yellow bars across the top line indicate degree of agreement among aligned sequences (green = 0% SNPs, yellow = presence of SNPs). In dark gray, a region of particularly low SNPs is highlighted.

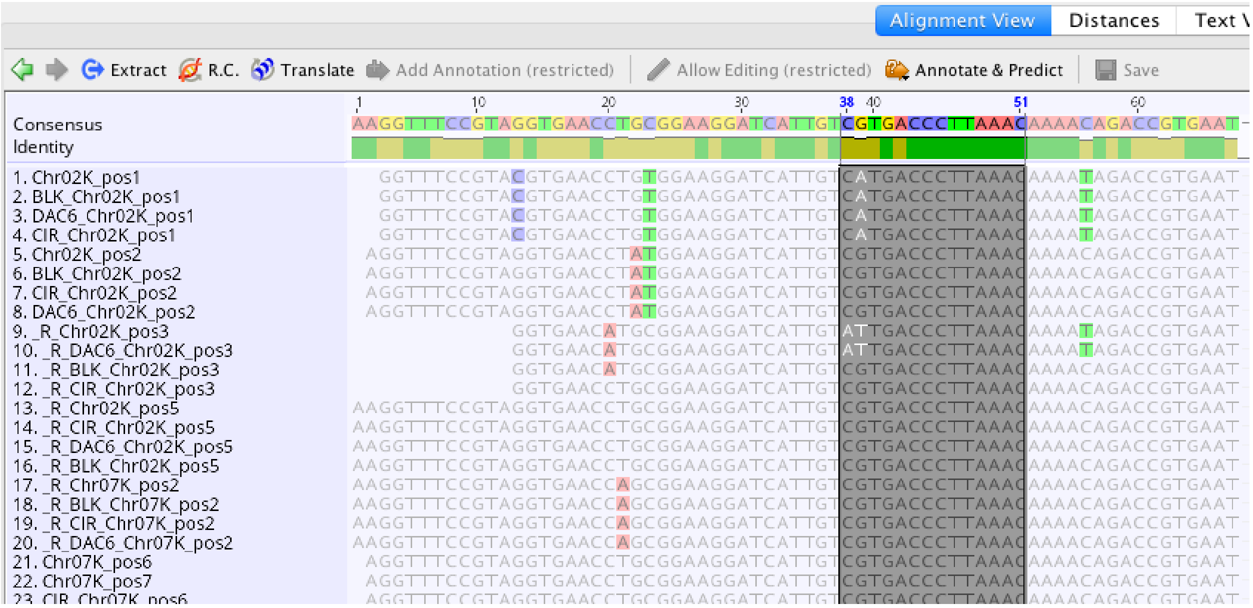

Figure S10 – Proportion of plant reads in amplicon sequencing varies by switchgrass variety and PNA concentration. Each point represents the proportion of plant reads in the Illumina dataset. Points are colored according to PNA concentration used during the first round of PCR, as in the legend. Violin plots show the distribution and range of plant reads for each switchgrass variety.

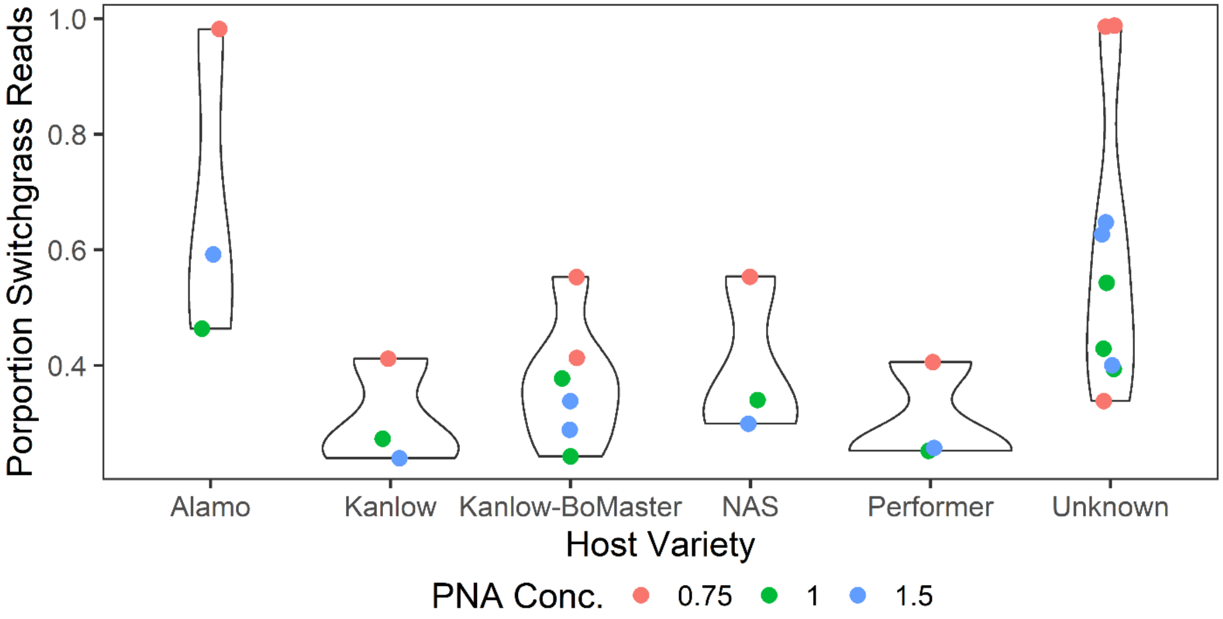

Figure S11 – Plant eukaryotic amplification (left panel) and sequencing depth (right panel) across crops. Each point represents a single sample. CRN = corn, SOY = soy, SWCH = switchgrass, WHT = wheat. The ‘control’ category includes both positive and negative controls.

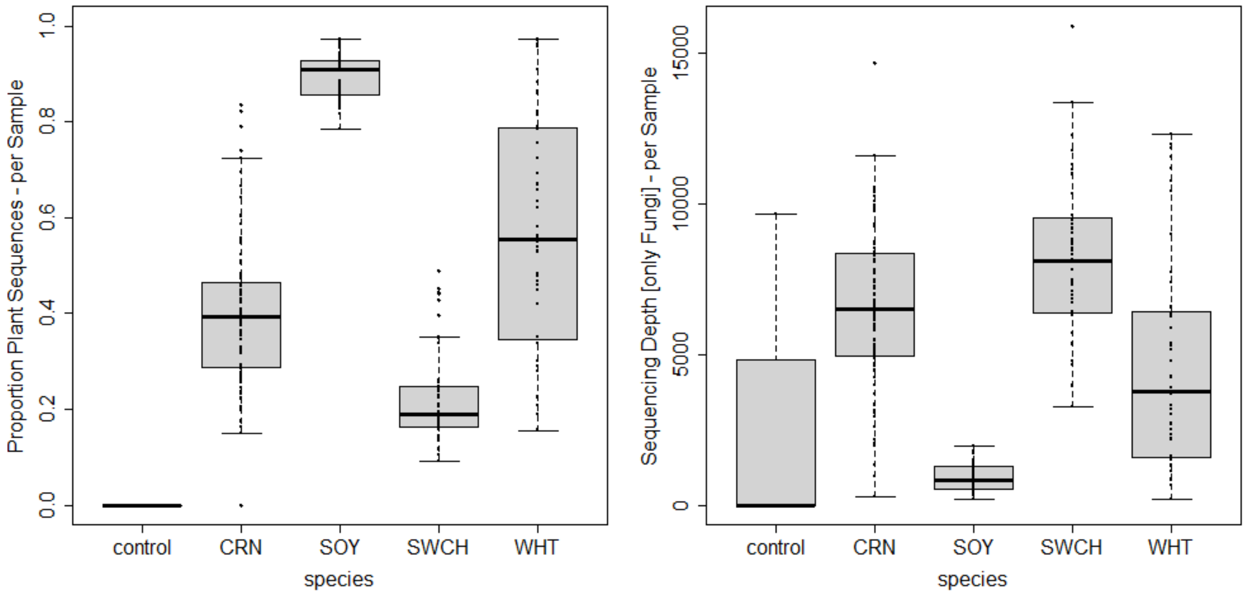

Figure S12 - Rarefaction curves for all samples. The x-axis has been shortened to 25,000 reads for visual simplicity.

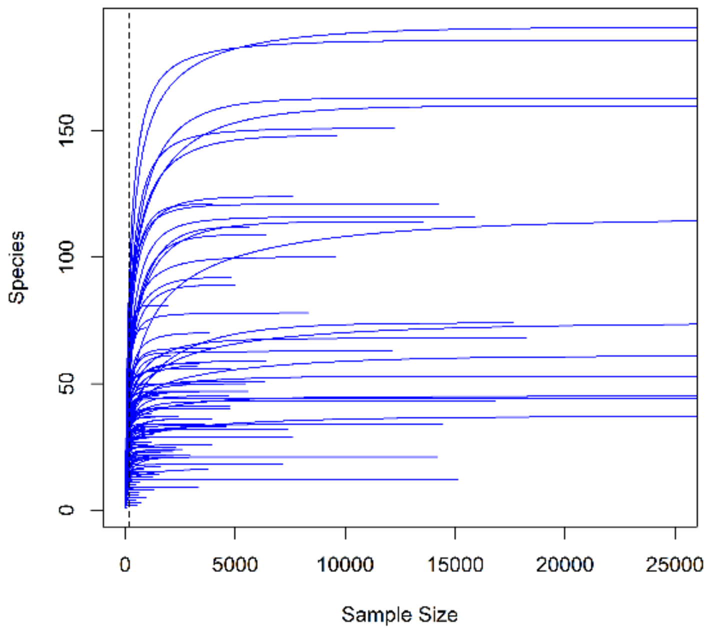

Figure S13 – Accumulation curves for each crop segregated by site: A) corn, B) soy, C), switchgrass, and D) wheat.

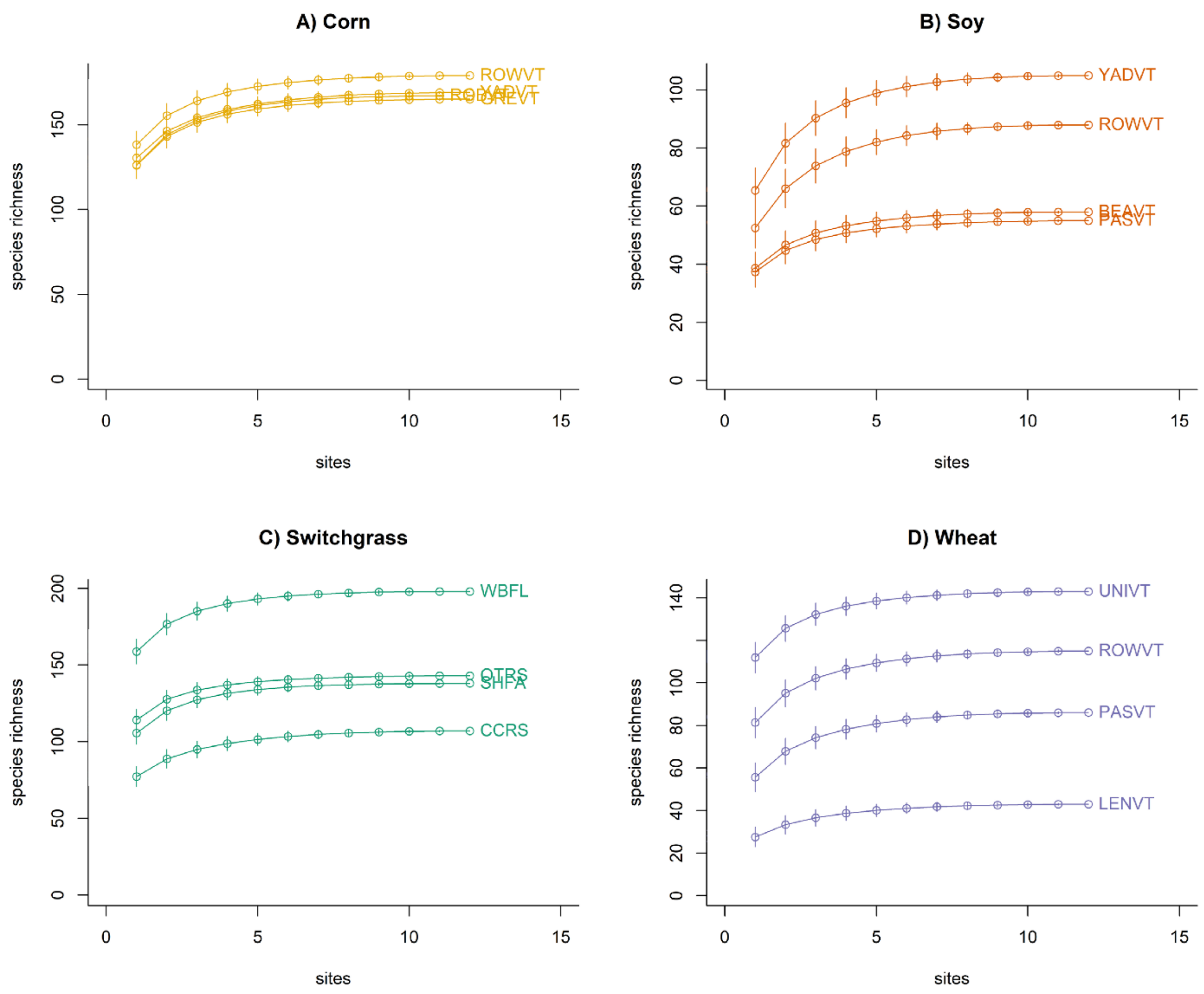

Table S1 – Previous crop rotation information for the 2019 sampled sites of corn, soy, and wheat, as well as the stand age information for the 2019 sampled sites of switchgrass.

**Crop in 2019**	**Site Abbreviation**	**Stand Age in 2019 (yrs)**	**Previous Crop**				
			**2018**	**2017**	**2016**	**2015**	**2014**
Corn	CRN-GREVT	*NA*	Sweet Potatoes	Soybeans	Corn	Sweet Potatoes	Soybeans
Corn	CRN-ROBVT	*NA*	Soybeans	Corn	Soybeans	Corn	Soybeans
Corn	CRN-ROWVT	*NA*	Soybeans/Wheat	Corn	Soybeans/Wheat	Corn	Soybeans/Wheat
Corn	CRN-YADVT	*NA*	Soybeans	Corn	Soybeans	Corn	Soybeans
Soy	SOY-BEAVT	*NA*	Corn	Soybeans	Corn	Soybeans	Corn
Soy	SOY-PASVT	*NA*	Cabbage	Corn	Soybeans	Cabbage	Corn
Soy	SOY-ROWVT	*NA*	Corn	Soybeans	Corn	Soybeans	Corn
Soy	SOY-YADVT	*NA*	Corn	Soybeans	Corn	Soybeans	Corn
Wheat	WHT-LENVT	*NA*	Corn	Soybeans/Wheat	Corn	Soybeans/Wheat	Corn
Wheat	WHT-PASVT	*NA*	Wheat/Soybeans	Wheat/Soybeans	Wheat/Soybeans	Wheat/Soybeans	Wheat/Soybeans
Wheat	WHT-ROWVT	*NA*	Corn	Soybeans/Wheat	Corn	Soybeans/Wheat	Corn
Wheat	WHT-UNIVT	*NA*	Soybeans	Wheat	Corn	Soybeans/Wheat	Corn
Switchgrass	SWCH-CCRS	27	*NA*	*NA*	*NA*	*NA*	*NA*
Switchgrass	SWCH-OTRS	9	*NA*	*NA*	*NA*	*NA*	*NA*
Switchgrass	SWCH-SHFA	2	*NA*	*NA*	*NA*	*NA*	*NA*
Switchgrass	SWCH-WBFL	8	*NA*	*NA*	*NA*	*NA*	*NA*

Table S2 - Variety information organized by crop species. Includes breeder or commercial owner, GMO traits, organization within the larger NCSU-OVT program, maturity information, and stress tolerance information collated from publically available variety trial information and from companies of origin. Stress tolerance information varied across crops and company information and are presented as follows, separated by semicolons (Environmental_Stress_Tolerance; Ear_Ratings; Soybean_Cyst_Nematode; Sudden_Death_Syndrome; Frogeye_Leaf_Spot; Stem_Canker; Root_Knot_Nematode; Reniform_Nematode; Phytopthora_Root_Rot; Pathogen_Tolerance).

Species	Variety	Sites Present	Company Brand	GMO Trait	GMO Trait Info	OVT Test Group 2019	Maturity	Stress Tolerance
CORN	66G25	CRN-GREVT, CRN-ROBVT, CRN-ROWVT, CRN-YADVT	Farmers Business Network (formerly Blue River Hybrids)	Conventional	2 MOA against corn-feeding insects	Medium	RM112	4 out of 5; 3.25 out of 5; NA; NA; NA; NA; NA; NA; NA; very good for everything, except Average for Gray Leaf Spot
CORN	LC1289	CRN-GREVT, CRN-ROBVT, CRN-ROWVT, CRN-YADVT	Local Seed	VT2PRIB	2 MOA against corn-feeding insects - not as good as RIB	Medium	RM112	Above Average; Above Average; NA; NA; NA; NA; NA; NA; NA; Very good NCLB, Average Gray Leaf Spot (N/A Rust & Anthracnose)
CORN	LC1586TC	CRN-GREVT, CRN-ROBVT, CRN-ROWVT, CRN-YADVT	Local Seed	TRECEPTA	hybrid of VT2P and DroughtGard	Medium	RM115	Above Average; Above Average; NA; NA; NA; NA; NA; NA; NA; very good for NCLB and Grey Leaf Spot, average Anthracnose (N/A Rust)
SOY	S13-10592C	SOY-BEAVT, SOY-PASVT, SOY-ROWVT, SOY-YADVT	University of Missouri	Conventional		MG4	4.5/4.6	chloine Excluder for salt tolerance; NA; NR; Resistant; Resistant; Resistant; NA; NA; NA; NA
SOY	MO4901D GT	SOY-BEAVT, SOY-PASVT, SOY-ROWVT, SOY-YADVT	VA Crop Improvement	RR		MG4	4.9	chloine Excluder for salt tolerance; NA; Moderate Resistance; Moderate Resistance; Resistant; Resistant; Moderate

								Resistance; Resistant; Field Tolerance; NA
SOY	S14-15146R	SOY-BEAVT, SOY-PASVT, SOY-ROWVT, SOY-YADVT	University of Missouri	RR/STS		MG4	4.6	NA; NA; NR; NA; Resistant; Resistant; NA; NA; NA; NA
WHEAT	Hilliard	WHT-LENVT, WHT-PASVT, WHT-ROWVT, WHT-UNIVT	VA-Tech [Griffey et al 2020 doi:10.1002/plr2.20073]			Experimenta l Wheat		NA; NA; NA; NA; NA; NA; NA; NA; NA; High yield NC 2018 (OVT data); resistant to many diseases
WHEAT	NC11363-25	WHT-LENVT, WHT-PASVT, WHT-ROWVT, WHT-UNIVT	NCSU			Experimenta l Wheat		NA; NA; NA; NA; NA; NA; NA; NA; NA; Med yield multi-state (SunTrials data); ranked well against powedery mildew, rusts, FHB
WHEAT	NC14-22588	WHT-LENVT, WHT-PASVT, WHT-ROWVT, WHT-UNIVT	NCSU			Experimenta l Wheat		NA; NA; NA; NA; NA; NA; NA; NA; NA; Low yield multi-state (SunTrials Data); ranked well against powdery mildew; ranked poorly against stripe rust; ranked med against FHB
SWITCH-GRASS	Performer	SWCH-CCRS, SWCH-WBFL, SWCH-OTRS	NCSU, USDA-ARS, NC-ARS					NA
SWITCH-GRASS	Kanlow	SWCH-SHFA, SWCH-OTRS	KAE, USDA-ARS					NA
SWITCH-GRASS	BoMaster	SWCH-SHFA	NCSU, USDA-ARS, NC-ARS					NA

Table S3 - Site metadata. Site and species codes are provided along with full site name, sampling date, location details, manager, soil type, leaf collection, and climate. All precipitation data are reported in mm and temperature data in °C. Elevation is in m.

Crop	Site Abbrev	Full Site Name	Lat	Lon	Elev	County	NC Region	Soil Type	Soil Texture	Precip 90day	LT 90day	HT 90day	MAP	MLT	MHT
CORN	CRN-GREVT	CORN Greene OVT	35.43000	-77.50331	9	Greene	Coastal Plains	Lumbee sandy loam	Sandy Loam	328.4	7.3	37.6	1234.1	10.5	22.8
CORN	CRN-ROBVT	CORN Robeson OVT	34.79335	-79.40648	64	Robeson	Piedmont	Wagram loamy sand	Loamy Sand	291.2	7.5	38.0	1159.5	10.1	23.1
CORN	CRN-ROWVT	CORN Rowan OVT	35.69863	-80.62482	220	Rowan	Piedmont	Lloyd clay loam	Clay Loam	311.5	6.6	34.9	1079.7	8.5	21.6
CORN	CRN-YADVT	CORN Yadkin OVT	36.47490	-80.64008	323	Yadkin	Mountains	Colvard and suches	Loam	487.6	4.7	34.0	1198.7	7.7	20.5
SOY	SOY-BEAVT	SOY Beaufort OVT	35.58162	-76.58198	3	Beaufort	Coastal Plains	Ponzer Muck	Organic	258.0	8.5	36.8	1256.1	10.9	22.5
SOY	SOY-PASVT	SOY Pasquotank-Perquimans OVT	36.26230	-76.25257	3	Pasquotank	Coastal Plains	Gertie silt loam	Silt Loam	352.4	8.8	36.8	1207.4	10.5	21.6
SOY	SOY-ROWVT	SOY Rowan OVT	35.69170	-80.62275	220	Rowan	Piedmont	Lloyd clay loam	Clay Loam	311.5	6.6	34.9	1079.7	8.5	21.6
SOY	SOY-YADVT	SOY Yadkin OVT	36.20152	-80.73361	343	Yadkin	Mountains	Clifford sandy clay loam	Sandy Clay Loam	431.3	4.9	33.4	1189.6	7.9	20.5
SWITCH-GRASS	SWCH-CCRS	SWITCHGRASS Central Crops Research Station	35.66531	-78.51256	100	Johnston	Piedmont	Wagram loamy sand	Loamy Sand	339.3	7.3	36.0	1177.0	9.5	21.8
SWITCH-GRASS	SWCH-OTRS	SWITCHGRASS Oxford Tobacco	36.30140	-78.61235	147	Granville	Piedmont	Helena/Vance	Loamy Sand	386.4	9.7	36.0	1127.6	8.6	21.1

		Research Station						sandy loam							
SWITCH-GRASS	SWCH-SHFA	SWITCHGRASS Shelby Farms	35.37303	-81.68670	300	Rutherford	Mountains	Pacolet/ Madison sandy clay loam/clay loam	Sandy Clay Loam/Clay Loam	386.7	6.7	35.0	1245.5	8.4	21.7
SWITCH-GRASS	SWCH-WBFL	SWITCHGRASS Williamsdale Biofuels Laboratory	34.76718	-78.10178	19	Duplin	Coastal Plains	Marvyn/Gritney/Noboco loamy fine sand	Loamy Fine Sand	243.4	10.5	37.4	1300.2	9.9	23.3
WHEAT	WHT-LENVT	WHEAT Lenoir OVT	35.37690	-77.56019	21	Lenoir	Coastal Plains	Lynchburg sandy loam	Sandy Loam	281.1	-4.2	30.5	1233.6	10.4	22.8
WHEAT	WHT-PASVT	WHEAT Pasquotank-Perquimans OVT	36.32312	-76.33748	5	Pasquotank	Coastal Plains	Wasda-Conaby complex, Roper Muck	Loam to Silt Loam	330.1	-5.5	30.8	1228.2	9.9	21.5
WHEAT	WHT-ROWVT	WHEAT Rowan OVT	35.69651	-80.62402	220	Rowan	Piedmont	Lloyd clay loam	Clay Loam	351.3	-5.0	29.6	1079.7	8.5	21.6
WHEAT	WHT-UNIVT	WHEAT Union OVT	35.13874	-80.37602	161	Union	Piedmont	Badin channery silt loam	Silt Loam	299.6	-3.3	30.7	1148.3	9.8	22.5

Table S4 - RRPP results for fungal a) community dissimilarity based on Euclidean distance and b) observed richness by crop species, site, and block.

a) Community dissimilarity

	df	SS	MS	R^2	*F*	Z	Pr(>F)
species	3	31980	10659.9	0.055302	8.3411	6.124	1.00E-04
species:site	12	52385	4365.4	0.090589	3.4158	13.4723	1.00E-04
species:site:block	47	60066	1278.0	0.103871	1.0931	5.317	1.00E-04
Residuals	128	149656	1169.2	0.258798			
Total	190	578273					

b) Richness

	df	SS	MS	R^2	*F*	Z	Pr(>F)
species	3	7802	2600.74	0.084855	50.7222	5.079	0.0001
species:site	12	6129	510.76	0.066658	9.961	6.013	0.0001
species:site:block	47	2410	51.27	0.026209	0.788	-0.929	0.8254
Residuals	128	8324	65.03	0.09053			
Total	190	91948					

Table S5 - RRPP results for fungal community distances by crop. a) Corn, b) Soy, c) Wheat, and d) Switchgrass. Factors tested include site, variety ("OVT.Variety"), and block.

a) Corn

	df	SS	MS	R^2	*F*	Z	Pr(>F)
site	3	7802	2601	0.0681	1.6340	NaN	0.0668
OVT.Variety	2	3230	1615	0.0282	1.0252	2.1099	0.0082
site:OVT.Variety	6	10014	1669	0.0874	1.0596	5.0608	0.0001
site:block	12	19100	1592	0.1667	0.0000	0.0000	0.5000
site:OVT.Variety:block	23	36228	1575	0.3163	0.0000	NaN	0.5008
Residuals	0	0	Inf	0			
Total	46	114550					

b) Soy

	df	SS	MS	R^2	*F*	Z	Pr(>F)
site	3	3278	1092.83	0.0649	1.6299	1.514	0.0684
OVT.Variety	2	1167	583.47	0.0231	0.7705	2.1732	0.0059
site:OVT.Variety	6	4859	809.86	0.0962	1.0694	4.6639	0.0001
site:block	12	8046	670.51	0.1593	NaN	NaN	0.6055
site:OVT.Variety:block	24	18174	757.27	0.3599	NaN	NaN	0.5394
Residuals	0	0	NaN	0			
Total	47	50500					

c) Wheat

	df	SS	MS	R^2	*F*	Z	Pr(>F)
site	3	4265	1421.81	0.0625	1.7133	1.5356	0.0785
OVT.Variety	2	970	485.01	0.0142	0.5843	2.1737	0.0043
site:OVT.Variety	6	4717	786.19	0.0691	0.9471	4.5998	0.0001
site:block	12	9958	829.86	0.1459	NaN	NaN	0.6306
site:OVT.Variety:block	24	19923	830.11	0.2919	NaN	NaN	0.6439
Residuals	0	0	NaN	0			
Total	47	68261					

d) Switchgrass

	df	SS	MS	R^2	*F*	Z	Pr(>F)
site	3	22626	7541.8	0.1557	4.1301	6.0442	0.0001
site:block	11	20087	1826.1	0.1383	1.2748	5.4744	0.0001
Residuals	33	47269	1432.4	0.3254			
Total	47	145288					

Table S6 - RRPP results for microbial community observed richness by crop. a) Corn, b) Soy, c) Wheat, and d) Switchgrass. Factors tested include site, variety ("OVT.Variety"), and block.

a) Corn

	df	SS	MS	R^2	*F*	Z	Pr(>F)
site	3	26.8	9	0.0075	0.1035	1.6131	0.1053
OVT.Variety	2	268.7	134	0.0753	1.7630	2.0998	0.0128
site:OVT.Variety	6	588.2	98	0.1649	1.2865	2.0223	0.0137
site:block	12	1034.0	86	0.2898	0.0000	0.0000	0.5000
site:OVT.Variety:block	23	1752.5	76	0.4912	0.0000	NaN	0.5161
Residuals	0	0.0	NaN	0.0000			
Total	46	3567.7					

b) Soy

	df	SS	MS	R^2	*F*	Z	Pr(>F)
site	3	8.8	2.92	0.0038	0.2000	1.6026	0.1088
OVT.Variety	2	20.7	10.33	0.0089	0.4934	2.1768	0.0179
site:OVT.Variety	6	146.8	24.47	0.0631	1.1684	2.1334	0.0108
site:block	12	175.0	14.58	0.0752	NaN	NaN	0.8870
site:OVT.Variety:block	24	502.7	20.94	0.2159	0	NaN	0.8469
Residuals	0	0.00	NaN	0.0000			
Total	47	2328.5					

c) Wheat

	df	SS	MS	R^2	*F*	Z	Pr(>F)
site	3	896	298.67	0.0738	8.5588	1.2795	0.0954
OVT.Variety	2	98.7	49.33	0.0081	2.0176	2.2006	0.0095
site:OVT.Variety	6	261.2	43.58	0.0215	1.7802	2.1902	0.0112
site:block	12	418.8	34.90	0.0345	NaN	NaN	0.9943
site:OVT.Variety:block	24	586.8	24.45	0.0483	NaN	NaN	0.7863
Residuals	0	0.0	NaN	0.0000			
Total	47	12147.0					

d) Switchgrass

	df	SS	MS	R^2	*F*	Z	Pr(>F)
site	3	3100.0	1033.34	0.2353	16.5656	3.5248	0.0008
site:block	11	686.2	62.38	0.0521	0.6869	-0.6250	0.7360
Residuals	33	2996.9	90.82	0.2275			
Total	47	13174.3					

Table S7 – The proportion of community structure explained by experimental factors and unexplained residual variation from variance partitioning analysis.

ID	Host Variety	Site	Block	Site and Block	Residual
Corn	0.00	0.12	0.02	0.24	0.64
Soy	0.01	0.11	0.00	0.20	0.70
Wheat	0.02	0.13	0.01	0.27	0.59
Switchgrass	N/A	0.16	0.03	0.34	0.46

Table S8 - CCA results (a,b,c) with WHT-PASVT site included (n=16 total), but soil characteristics excluded or (d,e,f) with WHT-PASVT site excluded (n=15 total) but soil characteristics included. Reported are: (a,d) ANOVA of CCA axes; (b,e) Permutation fit of environmental variables onto CCA ordination; (c,f) Loadings of environmental variables into CCA ordination.

a) ANOVA (n=16 sites)

	df	ChiSquare	*F*	Pr(>F)
CCA1	1	0.473	2.614	**0.041**
CCA2	1	0.379	2.096	0.245
CCA3	1	0.246	1.358	0.787
CCA4	1	0.190	1.052	0.974
CCA5	1	0.185	1.022	0.918
CCA6	1	0.153	0.846	0.892
CCA7	1	0.135	0.746	0.679
Residual	6	1.085		

b) Environmental fit (n=16 sites)

	CCA1	CCA2	r^2	Pr(>r)
Elev	0.979	0.204	0.002	0.992
Precip_90day	0.810	0.587	0.018	0.902
LT_90day	0.913	0.407	0.932	**0.001**
MAP	0.998	-0.064	0.064	0.661
Perc_veg_1km	0.954	0.299	0.044	0.784
soilmoist	-0.582	0.813	0.042	0.779
LAI.mean	0.834	-0.552	0.705	**0.001**

c) CCA Loadings (n=16 sites)

	CCA1	CCA2
Elev	0.049	0.008
Precip_90day	0.108	0.081
LT_90day	0.889	0.386
MAP	0.257	-0.030
Perc_veg_1km	0.208	0.060
soilmoist	-0.124	0.178
LAI.mean	0.713	-0.522

d) ANOVA (n=15 sites)

	df	ChiSquare	*F*	Pr(>F)
CCA1	1	0.490	3.144	**0.020**
CCA2	1	0.397	2.550	0.126
CCA3	1	0.234	1.502	0.791
CCA4	1	0.216	1.383	0.876
CCA5	1	0.183	1.174	0.972
CCA6	1	0.164	1.052	0.962
CCA7	1	0.158	1.014	0.923
CCA8	1	0.121	0.778	0.885
CCA9	1	0.112	0.718	0.620
Residual	3	0.468		

e) Environmental fit (n=15 sites)

	CCA1	CCA2	r^2	Pr(>r)
Elev	0.155	0.988	0.003	0.982
Precip_90day	0.093	0.996	0.016	0.929
LT_90day	0.409	0.912	0.806	**0.003**
MAP	0.371	0.929	0.121	0.495
Perc_veg_10km	0.994	0.112	0.483	**0.038**
soilmoist	-0.951	-0.308	0.045	0.784
LAI.mean	0.978	0.208	0.558	**0.015**
plantHt	0.236	0.972	0.501	**0.017**
pH	-0.775	-0.632	0.090	0.587

f) CCA Loadings (n=15 sites)

	CCA1	CCA2
Elev	0.008	0.054
Precip_90day	0.011	0.127
LT_90day	0.366	0.819
MAP	0.128	0.323
Perc_veg_10km	0.696	0.074
soilmoist	-0.203	-0.064
LAI.mean	0.735	0.151
plantHt	0.165	0.692
pH	-0.233	-0.188

Table S9 - Results from OLS models testing landcover predictors (a,b) with WHT-PASVT site included but soil characteristics excluded (n=16 sites) or (c,d) with WHT-PASVT site excluded but soil characteristics included (n=15 sites), for (a,c) average site observed richness and (b,d) average within-site dissimilarity. In all cases, the top five models are shown from the OLS best subsets model selection approach; best models based on Adj. R^2, AIC, and SBC are highlighted in bold. Predictors represent subset after accounting for multicolinearity (VIFs). Perc_veg_1km: % unmanaged vegetation 1-km scale; LT_90day: Low Temperature in preceeding 90 days; MAP: Mean Annual Precipitation (30-yr); LAI.mean: average site Leaf Area Index; pH: average site soil pH; soilmoist: average soil moisture; Precip_90day: Precipitation in preceeding 90 days.

a) Average Site Observed Richness (n=16 sites)

Model	R^2	Adj. R^2	AIC	SBC
Perc_veg_1km	0.2662	0.2138	143.27	145.59
**Perc_veg_1km + LT_90day**	**0.3840**	**0.2892**	**142.48**	**145.57**
Perc_veg_1km + LT_90day + MAP	0.4292	0.2865	143.26	147.12
Perc_veg_1km + LT_90day + MAP + LAI.mean	0.4374	0.2328	145.02	149.66
Perc_veg_1km + LT_90day + MAP + LAI.mean + soilmoist	0.4390	0.1585	146.98	152.39

b) Average Within-Site Dissimilarity (n=16 sites)

Model	R^2	Adj. R^2	AIC	SBC
Perc_veg_1km	0.3140	0.2650	119.48	121.8
**Perc_veg_1km + LT_90day**	**0.4183**	**0.3288**	**118.84**	**121.93**
Perc_veg_1km + LT_90day + Precip_90day	0.4464	0.3080	120.05	123.91
Perc_veg_1km + LT_90day + Precip_90day + MAP	0.4621	0.2665	121.59	126.22
Perc_veg_1km + LT_90day + Precip_90day + MAP + soilmoist	0.4680	0.2020	123.41	128.82

c) Average Site Observed Richness (n=15 sites)

Model	R^2	Adj. R^2	AIC	SBC
Perc_veg_1km	0.2197	0.1597	135.66	137.79
**Perc_veg_1km + LT_90day**	**0.3871**	**0.2850**	**134.04**	**136.87**
Perc_veg_1km + LT_90day + MAP	0.4288	0.2730	134.98	138.52
Perc_veg_1km + LT_90day + MAP + LAI.mean	0.4384	0.2137	136.73	140.98
Perc_veg_1km + LT_90day + MAP + LAI.mean + pH	0.4431	0.1337	138.60	143.56

d) Average Within-Site Dissimilarity (n=15 sites)

Model	R^2	Adj. R^2	AIC	SBC
Perc_veg_1km	0.2566	0.1994	113.35	115.48
**Perc_veg_1km + LT_90day**	**0.4014**	**0.3016**	**112.10**	**114.94**
Perc_veg_1km + LT_90day + Precip_90day	0.4301	0.2747	113.36	116.90
Perc_veg_1km + LT_90day + Precip_90day + MAP	0.4437	0.2212	115.00	119.25
Perc_veg_1km + LT_90day + Precip_90day + MAP + soilmoist	0.4540	0.1507	116.72	121.68

Table S10 – Results from mixed effects regression models testing the significant site-level effect of percent unmanaged vegetation at the plot level for: (a) observed richness and (b) plot dissimilarity. Crop species were tested as random intercepts and as combined random intercepts and slopes. Percent unmanaged vegetation at the 1-km scale was the independent variable tested.

a) Observed Richness

Model	*P*	AIC
Random Intercepts	<0.001	1534.0
Random Intercepts and Slopes	<0.001	1538.0

b) Plot Dissimilarity

Model	*P*	AIC
Random Intercepts	<0.001	1266.8
Random Intercepts and Slopes	0.005	1267.2

Table S11 - USDA NASS Cropland Datalayers (CDL). Two tables are provided in the CDL, the cropland and cultivated tables. The cropland table was used for manual annotation of all landcover types into one of three landcover classes for analyses: 1) crop, 2) other/unmanaged vegetation, and 3) non-vegetation/ non-crop.

**Cropland classes**				
**Value**	**Color**	**Description**	**Manual_Annotation**	**Notes**
1	ffd300	Corn	crop	
2	ff2626	Cotton	crop	
3	00a8e2	Rice	crop	
4	ff9e0a	Sorghum	crop	
5	267000	Soybeans	crop	
6	ffff00	Sunflower	crop	
10	70a500	Peanuts	crop	
11	00af49	Tobacco	crop	
12	dda50a	Sweet Corn	crop	
13	dda50a	Pop or Orn Corn	crop	
14	7cd3ff	Mint	crop	
21	e2007c	Barley	crop	
22	896054	Durum Wheat	crop	
23	d8b56b	Spring Wheat	crop	
24	a57000	Winter Wheat	crop	
25	d69ebc	Other Small Grains	crop	
26	707000	Dbl Crop WinWht/Soybeans	crop	
27	aa007c	Rye	crop	
28	a05989	Oats	crop	
29	700049	Millet	crop	
30	d69ebc	Speltz	crop	
31	d1ff00	Canola	crop	
32	7c99ff	Flaxseed	crop	
33	d6d600	Safflower	crop	
34	d1ff00	Rape Seed	crop	
35	00af49	Mustard	crop	
36	ffa5e2	Alfalfa	crop	

37	a5f28c	Other Hay/Non Alfalfa	crop	*may not ~ALWAYS be considered cultivated in 0/1 cultivated dataset
38	00af49	Camelina	crop	
39	d69ebc	Buckwheat	crop	
41	a800e2	Sugarbeets	crop	
42	a50000	Dry Beans	crop	
43	702600	Potatoes	crop	
44	00af49	Other Crops	crop	
45	af7cff	Sugarcane	crop	
46	702600	Sweet Potatoes	crop	
47	ff6666	Misc Vegs & Fruits	crop	
48	ff6666	Watermelons	crop	
49	ffcc66	Onions	crop	
50	ff6666	Cucumbers	crop	
51	00af49	Chick Peas	crop	
52	00ddaf	Lentils	crop	
53	54ff00	Peas	crop	
54	f2a377	Tomatoes	crop	
55	ff6666	Caneberries	crop	
56	00af49	Hops	crop	
57	7cd3ff	Herbs	crop	
58	e8bfff	Clover/Wildflowers	crop	very minor contributer in NC
59	afffdd	Sod/Grass Seed	crop	*may not ~ALWAYS be considered cultivated in 0/1 cultivated dataset
60	00af49	Switchgrass	crop	
61	bfbf77	Fallow/Idle Cropland	other/unmanaged vegetation	
63	93cc93	Forest	other/unmanaged vegetation	
64	c6d69e	Shrubland	other/unmanaged vegetation	
65	ccbfa3	Barren	not-vegetation/non-crop	
66	ff00ff	Cherries	crop	
67	ff8eaa	Peaches	crop	
68	ba004f	Apples	crop	
69	704489	Grapes	crop	

70	7777	Christmas Trees	crop	
71	af9970	Other Tree Crops	crop	
72	ffff7c	Citrus	crop	
74	b5705b	Pecans	crop	
75	00a582	Almonds	crop	
76	e8d6af	Walnuts	crop	
77	af9970	Pears	crop	
81	f2f2f2	Clouds/No Data	not-vegetation/non-crop	*basically not an issue, tested most of NC and coming out with 0.0000 %
82	999999	Developed	not-vegetation/non-crop	
83	4970a3	Water	not-vegetation/non-crop	
87	7cafaf	Wetlands	not-vegetation/non-crop	
88	e8ffbf	Nonag/Undefined	not-vegetation/non-crop	
92	00ffff	Aquaculture	not-vegetation/non-crop	
111	4970a3	Open Water	not-vegetation/non-crop	
112	d3e2f9	Perennial Ice/Snow	not-vegetation/non-crop	
121	999999	Developed/Open Space	not-vegetation/non-crop	
122	999999	Developed/Low Intensity	not-vegetation/non-crop	
123	999999	Developed/Med Intensity	not-vegetation/non-crop	
124	999999	Developed/High Intensity	not-vegetation/non-crop	
131	ccbfa3	Barren	not-vegetation/non-crop	
141	93cc93	Deciduous Forest	other/unmanaged vegetation	
142	93cc93	Evergreen Forest	other/unmanaged vegetation	
143	93cc93	Mixed Forest	other/unmanaged vegetation	
152	c6d69e	Shrubland	other/unmanaged vegetation	
176	e8ffbf	Grassland/Pasture	other/unmanaged vegetation	
190	7cafaf	Woody Wetlands	other/unmanaged vegetation	
195	7cafaf	Herbaceous Wetlands	other/unmanaged vegetation	
204	00ff8c	Pistachios	crop	
205	d69ebc	Triticale	crop	
206	ff6666	Carrots	crop	
207	ff6666	Asparagus	crop	
208	ff6666	Garlic	crop	

209	ff6666	Cantaloupes	crop	
210	ff8eaa	Prunes	crop	
211	334933	Olives	crop	
212	e27026	Oranges	crop	
213	ff6666	Honeydew Melons	crop	
214	ff6666	Broccoli	crop	
215	739755	Avocados	crop	
216	ff6666	Peppers	crop	
217	af9970	Pomegranates	crop	
218	ff8eaa	Nectarines	crop	
219	ff6666	Greens	crop	
220	ff8eaa	Plums	crop	
221	ff6666	Strawberries	crop	
222	ff6666	Squash	crop	
223	ff8eaa	Apricots	crop	
224	00af49	Vetch	crop	
225	ffd300	Dbl Crop WinWht/Corn	crop	
226	ffd300	Dbl Crop Oats/Corn	crop	
227	ff6666	Lettuce	crop	
228	f8d248	Dbl Crop Triticale/Corn	crop	
229	ff6666	Pumpkins	crop	
230	896054	Dbl Crop Lettuce/Durum Wht	crop	
231	ff6666	Dbl Crop Lettuce/Cantaloupe	crop	
232	ff2626	Dbl Crop Lettuce/Cotton	crop	
233	e2007c	Dbl Crop Lettuce/Barley	crop	
234	ff9e0a	Dbl Crop Durum Wht/Sorghum	crop	
235	ff9e0a	Dbl Crop Barley/Sorghum	crop	
236	a57000	Dbl Crop WinWht/Sorghum	crop	
237	ffd300	Dbl Crop Barley/Corn	crop	
238	a57000	Dbl Crop WinWht/Cotton	crop	
239	267000	Dbl Crop Soybeans/Cotton	crop	
240	267000	Dbl Crop Soybeans/Oats	crop	
241	ffd300	Dbl Crop Corn/Soybeans	crop	

242	..000099	Blueberries	crop	
243	ff6666	Cabbage	crop	
244	ff6666	Cauliflower	crop	
245	ff6666	Celery	crop	
246	ff6666	Radishes	crop	
247	ff6666	Turnips	crop	
248	ff6666	Eggplants	crop	
249	ff6666	Gourds	crop	
250	ff6666	Cranberries	crop	
254	267000	Dbl Crop Barley/Soybeans	crop	

**Cultivated Classes**				
**Value**	**Color**	**Description**		
1	d3d3d3	Non-cultivated		
2	b1b58c	Cultivated		

Table S12 - Results of switchgrass K-mers tested for design specifications with PNABio. Originally selected from within the first 100bp of forward primer in the ITS1, with few or no SNPS to switchgrass ITS genomic sequences.

kmer_number	kmer_seq	Tm	Purine_Perc	GC_content	Design	Self_complementarity	complementarity_notes
kmer_1	ATTGTCGTGACCC	67.5	38.5	53.8	Good	some	
kmer_2	TTGTCGTGACCCT	67.1	30.8	53.8	Good	some	
kmer_3	TGTCGTGACCCTT	67.1	30.8	53.8	Good	some	
kmer_4	GTCGTGACCCTTA	66.2	38.5	53.8	Good	some	
kmer_5	TCGTGACCCTTAA	65.4	38.5	46.2	Good		
kmer_6	CGTGACCCTTAAA	66.1	46.2	46.2	Good		
kmer_7	GTGACCCTTAAAC	64.1	46.2	46.2	Good		
kmer_10	ACCCTTAAACAAA	64.5	53.8	30.8	BAD		
kmer_12	ATTGTCGTGACCCT	69.4	35.7	50.0	Good	some	
kmer_13	TTGTCGTGACCCTT	67.7	28.6	50.0	Good	some	
kmer_14	TGTCGTGACCCTTA	68.5	35.7	50.0	Good	some	
kmer_15	GTCGTGACCCTTAA	68.8	42.9	50.0	Good	some	
kmer_16	TCGTGACCCTTAAA	68.0	42.9	42.9	Good		
kmer_17	CGTGACCCTTAAAC	67.5	42.9	50.0	Good		
kmer_22	ATTGTCGTGACCCTT	69.8	33.3	46.7	Good	some	
kmer_23	TTGTCGTGACCCTTA	69.0	33.3	46.7	Good	some	
kmer_24	TGTCGTGACCCTTAA	70.8	40.0	46.7	Good	some	
kmer_25	GTCGTGACCCTTAAA	71.0	46.7	46.7	Good	some	
kmer_26	TCGTGACCCTTAAAC	69.2	40.0	46.7	Good		
kmer_31	ATTGTCGTGACCCTTA	71.0	37.5	43.8	Good	some	
kmer_32	TTGTCGTGACCCTTAA	71.2	37.5	43.8	Good	some	
kmer_33	TGTCGTGACCCTTAAA	72.8	43.8	43.8	Good	some	
kmer_34	GTCGTGACCCTTAAAC	72.0	43.8	50.0	Good	some	
kmer_39	ATTGTCGTGACCCTTAA	72.9	41.2	41.2	Good	some	
kmer_40	TTGTCGTGACCCTTAAA	73.1	41.2	41.2	Good	some	TTgtcGTgacCCTTAAA
kmer_41	TGTCGTGACCCTTAAAC	73.7	41.2	47.1	Good	some	TgtcGTgacCCTTAAAC

SUPPLEMENTARY METHODS

Switchgrass sampling design across sites

To sample switchgrass, we attempted to match the design of the OVT trials as closely as possible. In sites with large untreated switchgrass fields (CCRS, SHFA), plot locations were assigned in a stratified random design to mimic the 4 blocks used in the OVT trials, with 3 plots per block. In sites where we sampled existing switchgrass experiments (OTRS, WBFL), we selected a balanced design with 3 or 4 blocks that accommodated existing treatments (with 4 or 3 plots per block respectively for 12 total plots per site). Sampling from switchgrass plots always included at least a 1.5-m border to avoid edge effects. Switchgrass cultivars were not replicated across the sites. Details on sampling at specific sites are listed below.

The SWCH-CCRS is managed by NC Department of Agriculture and Consumer Services (NCDA&CS) and contains a single stand of Performer variety switchgrass that is mowed and burned annually in the spring, but is not harvested. The SWCH-SHFA is independently maintained by a farmer in NC and contains a single large stand with a mix of Kanlow and BoMaster varieties. SWCH-SHFA is harvested annually for livestock feed. Blocks were randomly assigned across both of these large stands as described above.

The SWCH-OTRS site is managed by NCSU and included a fertilizer by variety management treatment with no replication. Blocks were constructed according to the spatial layout of the field, crossing management treatments. We constructed two plots within each of two varieties (Kanlow and Performer) and for all 3 fertilizer treatments (none, low, high), creating 12 plots total. Fertilizer levels were applied according to treatment (0, 6.73, or 13.46 g N m^{-2} yr^{-1}). SWCH-OTRS is neither harvested nor mowed/burned.

The SWCH-WBFL site is managed by NCSU and contains three replicate blocks of switchgrass (Performer Variety) with fertilizer by harvest by biofuel management treatments as subplots. We sampled exclusively from the two lowest fertilizer treatments (0 and 6.73 g N m^{-2} yr^{-1}) crossed with the two single-harvest treatments of an October harvest and an October harvest with rye winter cover crop. Thus, for three blocks and four management treatment subplots, we sampled n=12 total plots. From each plot, we sampled an area measuring 2.4 m x 4.9 m to avoid edge effects and to approximate the area of plots sampled at other sites.

Details on plant, soil, and vegetative cover measurements

Plot-level latitude and longitude were recorded at the center point of each plot using a handheld GPS (Garmin International, Inc., Olathe, KS, USA).

Plant height and LAI may increase niche space for colonizing/dispersing foliar fungi (Meyer, et al. 2022) or may reflect an altered plant-microbiome relationship due to the linkages of plant performance to overall plant health and nutrition (Christian, et al. 2019, Whitaker, et al. 2015). Additionally, plant height and LAI were used as proxies for plant performance because they predicted vegetative yield in switchgrass (Aspinwall, et al. 2017)and because height was strongly correlated with grain yields (Site-wide average Yield ~ Height, =11; Adj. R^2=0.92).

Plant height was measured on four plants within a plot and then averaged to get an average plot height. For wheat, corn, and switchgrass, height was measured at the point where the flag leaf met the tiller. For soybean, height was measured at the point where three leaflets met the petiole of the tallest fully unfurled leaf.

For each plant, leaf area index values (LAI, m^2 m^{-2}) were estimated from ceptometer (Accupar, LP-80, Decagon Devices Inc., Pullman, WA) measurements at 5 cm height, taken in

four directions spanning 180 degrees through the crown of each plant. For corn plots, only half of the ceptometer was used (40 cm) to reduce edge effects caused by adjacent plots (i.e., corn plots were each two-rows and more densely planted). Unless otherwise stated, for corn, soy, and wheat sites average plot LAI was calculated from the LAI measurements of four plants per plot. For the switchgrass sites, average plot LAI was constructed from the average of two plants, while at CRN-YADVT, it was constructed from one centrally located plant. The ceptometer provided raw data on the above (A) and below (B) canopy PAR. Beer's law (Flenet, et al. 1996) was applied to the fraction of intercepted light (FIPAR = [A-B]/A) to correct for the light extinction coefficient of the canopy (or K). Thus:

$$LAI = [\ln(1-FIPAR)]/-K$$

K values were chosen for each crop to mimic known row spacing, fertilization, and climatic conditions. K values were as follows: corn = 0.43, soybean = 0.49, (Flenet, et al. 1996), switchgrass = 0.33 (Kiniry, et al. 2011, Kiniry, et al. 1999), and wheat = 0.56 (Li, et al. 2021, Pradhan, et al. 2018).

Soil moisture was measured in one of two ways. For wheat, soil moisture was measured for each plot using a handheld probe and sensor (HH2 and ML3, Dynamax Inc., Houston, TX), with one reading taken per each of four plants per plot and averaged at the plot level. For corn, soybean, and switchgrass, soil moisture was measured by soil gravimetrics. Specifically, one core per each of four plants was collected and bulked at the plot level. Cores were collected using a trowel to a 7 cm depth. Soil collected in the field was stored in a sealed bag at 4ºC for four days, fresh weight measured, oven dried at 105 C for 48hrs, then dry weight measured. Then, soil moisture was computed as the percentage water loss from fresh to dry weight. All soil moisture measurements were collected within 13 cm of the plant crown.

Soil chemical and physical properties were averaged at the site level and method of collection varied among crops. For wheat sites, three soil cores to 15 cm depth within 8 cm of wheat crowns were collected in May 2019 and analyzed by the NCDA&CS to determine site-level values. For corn and soy sites, the soil collected for the soil moisture measurements was air dried, analyzed by the NCDA&CS, and averaged for site-level soil properties. For switchgrass, site-level averages were calculated for soils collected in 2018 and analyzed as reported in Lee & Hawkes (Lee and Hawkes 2021). Data from the corn and soy sites showed that the coefficient of variation among plots for the soil properties was low (pH: 0.79-1.21%; HM 0.46-19.5%).

To calculate percentage of umanaged natural or semi-natural vegetation and active cropland, we first manually assigned each landcover class to one of three categories: 1) cropland/managed, 2) unmanaged/natural and semi-natural vegetation, 3) non-cropland/non-vegetation or otherwise urban spaces (Supplementary Table S11). For each site, percent unmanaged vegetation and percent cropland were calculated for the surrounding 1-km and 10-km radii (Supplementary Fig. S2A-E). Across all crop species and sites, percent unmanaged vegetation ranged from 0.2-53% at the 1-km scale and 27-72% at the 10-km scale, while percent cropland cover ranged from 13-99% and 10-60% at the 1-km and 10-km scales, respectively (Supplementary Fig. S3). Cover of unmanaged vegetation and cropland were negatively correlated (r^2 = 0.49-0.77; Supplementary Fig. S2F).

Illumina library preparation and bioinformatics

DNA concentration was determined using AccuClear Ultra High Sensitivity dsDNA (Biotium Inc., Fremont, CA) on a Synergy H1 microplate reader (one technical replicate, 468/507 ex/em; Agilent Technologies, Santa Clara, CA) and normalized to 20 ng μL^{-1}. Gene

amplification of DNA template was performed using two-stage PCR with modified versions of the ITS1F (5’ CTTGGTCATTTAGAGGAAGTAA 3’) and ITS2 (5’ GCTGCGTTCTTCATCGATGC 3’) fungal primers (Smith and Peay 2014). Positive and negative PCR controls were included in each stage of amplification. First stage PCR was run in duplicate for all samples. Each reaction consisted of 12.5 µL 1× KAPA HiFi HotStart ReadyMix (Roche Sequencing Solutions, Indianapolis, IN), 0.2 µM of each primer, 0.5 mg mL^{-1} BSA, 20 ng DNA template, and PCR-grade water up to 25 µL. For the switchgrass samples, 1.5 µM of a custom-designed peptide nucleic acid (PNA) was added to the PCR reaction in lieu of water to block host genomic ITS amplification (see ‘Switchgrass PNA design and testing’ section below for more details; Fig. S9, Fig. S10, and Table S12). Thermocycling conditions were 45 s at 98°C, followed by 25 cycles of denaturing for 15 s at 98°C, annealing for 30 s at 58 C, and extension for 30 s for 72°C, and a final extension for 5 min at 72°C. For the switchgrass samples, an additional PNA annealing step of 10 s at 70°C was included prior to primer annealing.

Technical replicates were pooled and cleaned using AMPure XP beads (Beckman-Coulter Inc., Indianapolis, IN). Each index PCR reaction consisted of 12.5 µL 1× KAPA HiFi HotStart ReadyMix, 2.5 µL each of two indices (Nextera XT Index Kit v2, Illumina Inc., San Diego, CA), 2.5 µL DNA template, and 5 µL water. All products were cleaned using AMPure XP beads, quantified in triplicate using AccuClear on a Synergy H1 Microplate reader (468/507 ex/em), and PCR fragment size determined using Tapestation (D1000 ScreenTape, Agilent Technologies, Santa Clara, CA). Lastly, all samples were pooled in equimolar ratios and sequenced on an Illumina MiSeq platform (v3, 2 x 300bp, paired-end) at NCSU’s High Performance Computing center.

Primers were removed using cutadapt (v.1.18; Martin 2011) and default parameters were used for all other steps, except at the filtering stage (maxEE = 2,4 – which was chosen to optimize read counts per sample and benefit rare ASV recovery). Plant ASVs were screened using BLAST matches (-word_size 50, -perc_identity 60) to a manually curated database of both full length ITS sequences for all plant species sampled (47 GenBank sequences) and the UNITE database (sh_general_release_dynamic_02.02.2019.fasta; (Kõljalg, et al. 2013). For sequences with neither high confidence matches to plants nor fungi in the first BLAST, a second screening process was applied by BLAST-ing the NCBI nr database (Clark, et al. 2016). After filtering, denoising, merging, chimera check, and plant sequence removal, the raw paired read count of experimental samples was reduced from 13,670,276 to 1,002,267. Note that additional samples were included in the MiSeq run and bioinformatics processing that were not analyzed here.

Fungal sequences were further curated with the co-occurrence based, post-clustering method, LULU, using default parameters (v.0.1.0; Frøslev, et al. 2017); this method identifies “daughter” ASVs that are fragments of and consistently co-occur with “parent” ASVs and merges them, thereby reducing artefactual ASV designations. Analysis of the positive and negative controls revealed that two reads of a legitimate ASV were present in one negative control and two reads of the positive control (a pure *Biscogniauxia* sp. originally isolated in Texas) were present in one experimental sample. Thus, we removed ASVs with fewer than 2 reads (as the level of non-target sequence contamination) or found in only one sample (i.e. singletons), resulting in 595 ASVs. Putative names were assigned using the RDP naïve Bayesian classifier (Wang, et al. 2007). The Warcup fungal ITS database (v2; Deshpande, et al. 2016) was used as the default with substitutions from the UNITE fungal ITS database (Kõljalg, et al. 2013) for all taxa with confidence scores below 70%. Fungal richness (vegan v.2.6-2; Oksanen, et al. 2022) was calculated after quality filtering. Soybean samples experienced particularly high plant

eukaryotic read amplification (Supplementary Fig. S11). However, rarefaction curves per sample and accumulation curves at the site level were asymptotic (Supplementary Fig. S12-S13), indicating sufficient sampling for diversity analyses. The use of the variance stabilizing transformation also regularized the mean-variance relationship across all samples (McMurdie and Holmes, 2014). All bioinformatics R scripts are archived on Zenodo (Whitaker 2023).

Switchgrass PNA design and testing

To reduce non-target switchgrass ITS amplification, we designed a peptide nucleic acid (PNA) blocker using design parameters identified in (Cregger, et al. 2018, Lundberg, et al. 2013), including melting temperature higher than the relevant primer set, low self-complementarity, adequate dissolvability, and within 100 bp of the forward primer in the region of interest. To design a PNA for the ITS1 region in switchgrass, we borrowed an approach from the field of comparative genomics to determine natural ITS variation both intra- and inter-genomically. First, the AP13 reference genome (v.5.0; Lovell, et al. 2021) was imported from JGI Phytozome (Goodstein, et al. 2012) into the Galaxy web platform for further processing using the public server (https://usegalaxy.org/; Afgan, et al. 2018). Six ITS sequences of common switchgrass varieties were downloaded from NCBI's nr database and blasted against the AP13 reference genome (word size = 11, % identity cutoff = 90), then used to create a BED file, and the ITS sequences extracted from the AP13 genome in fasta format (bedtools, v.2.29.0; Quinlan and Hall 2010).

Simultaneously, three resequenced genomes of different switchgrass varieties, including two upland and one lowland (DAC6, Blackwell_WO1, Cave_IN_Rock_WO1; PIs Daniel Rokhsar, Thomas E. Juenger, David Lowry, Laura Elizabeth Bartley), were imported into Galaxy from JGI's Genome Portal (Nordberg, et al. 2014). Each genome was groomed, quality filtered (to 50% of the read with aggregate quality >= 30), mapped to the AP13 reference genome ITS sequences using BWA-MEM (v.0.7.17; Li and Durbin 2010), and sorted by coordinates using SAMtools (v.1.9; Li, et al. 2009). Next, bcftools (v.1.9; Li, et al. 2009) was used to produce genotype likelihoods (mpileup; 250 max reads per BAM), call variants (call), normalize indels (norm), filter variants (filter; adjacent indels <= 5pb and quality scores >= 10), and create consensus sequences in fasta format (consensus). Finally, the sequence variants across all four genomes, including the reference, were aligned using MAFFT (LINSI flavor, v.7.221; Katoh and Standley 2013).

Regions within the first 100 bp of the forward primer with few to no SNPs were broken into k-mers of 13-17 bp lengths (Supplementary Fig. S9), which were then blasted against the UNITE fungal database (Kõljalg, et al. 2013). K-mers with no fungal matches (Supplementary Table S12) were imported into PNABio (https://www.pnabio.com/products/PNA_oligo.htm) and the PNA with the most optimal design specifications selected for laboratory tests (kmer_41, 5'-TGTCGTGACCCTTAAAC-3'; Supplementary Fig. S9).

To determine PNA effectiveness, we tested three different PNA concentrations (0.75 μM, 1.0 μM, and 1.5 μM) and four switchgrass varieties, as well as samples from a mixed stand of two varieties, and two unknown varietal samples. PCR conditions, as well as purification, indexing, and sequencing were as described in the Methods, except that Illumina sequencing was performed using a Nano v2 PE chip (Illumina Inc., San Diego, CA). Previous research had shown switchgrass read contamination as high as 95% in the ITS1 (Whitaker unpublished). The proportion of non-target switchgrass reads varied across known and unknown switchgrass varieties (P = 0.006) and across PNA concentrations (P = 0.003; tested by 2-factor ANOVA,

Type III SS; Supplementary Fig. S10). A Tukey's Honest Significant Difference test showed that both 1.0 μM and 1.5 μM PNA similarly reduced switchgrass more than 0.75 μM PNA (both $P < 0.05$) and were not statistically different from one another ($P = 0.82$). It was not possible to test the interaction between variety and PNA concentration. However, visual inspection of the data indicated that the highest PNA concentration tested (1.5 μM) was generally more effective at reducing switchgrass amplification in known switchgrass varieties (i.e., Kanlow, NAS, Kanlow-BoMaster mix, and Performer).

FUNGuild assignment of Pathotroph community

We used FUNGuild to assign all ASVs to one of three functional groups: pathotroph, saprotroph, and symbiotroph (Nguyen, et al. 2016). ASVs assigned to multiple groups (e.g., pathotroph-symbiotroph) or with only a "possible" confidence assignment were excluded (Lozano, et al. 2021). This approach allowed for 35% of ASV assignments covering 51% of the sequencing reads; approximately half were pathotrophs. Given the limited number of confident assignments overall, we only analyzed the pathotroph data for trends to assess similarity to the full dataset. Thus, we modelled the average within-site proportion of pathotroph sequencing reads, and the standard error, as a function of the same explanatory variables included in regressions of richness and dissimilarity of the full fungal community. The best models were determined using Akaike Information Criteria (AIC), Schwarz Bayesian Criteria (SBC), and Adjusted R^2 (olsrr::ols_step_best_subset; Hebbali 2020).

REFERENCES FOR SUPPLEMENTARY METHODS

Afgan E, Baker D, Batut B *et al.* (2018) The Galaxy platform for accessible, reproducible and collaborative biomedical analyses: 2018 update. *Nucleic Acids Res* **46**: W537-W44.

Aspinwall MJ, Fay PA, Hawkes CV *et al.* (2017) Intraspecific variation in precipitation responses of a widespread C_4 grass depends on site water limitation. *J Plant Ecol* **10**: 310-21.

Christian N, Herre EA, Clay K (2019) Foliar endophytic fungi alter patterns of nitrogen uptake and distribution in *Theobroma cacao*. *New Phytologist* **222**: 1573-83.

Clark K, Karsch-Mizrachi I, Lipman DJ *et al.* (2016) GenBank. *Nucleic Acids Res* **44**: D67-D72.

Cregger MA, Veach AM, Yang ZK *et al.* (2018) The *Populus* holobiont: dissecting the effects of plant niches and genotype on the microbiome. *Microbiome* **6**: 1-14.

Deshpande V, Wang Q, Greenfield P *et al.* (2016) Fungal identification using a Bayesian classifier and the Warcup training set of internal transcribed spacer sequences. *Mycologia* **108**: 1-5.

Flenet F, Kiniry JR, Board JE *et al.* (1996) Row spacing effects on light extinction coefficients of corn, sorghum, soybean, and sunflower. *Agron J* **88**: 185-90.

Frøslev TG, Kjøller R, Bruun HH *et al.* (2017) Algorithm for post-clustering curation of DNA amplicon data yields reliable biodiversity estimates. *Nat Commun* **8**: 1188.

Goodstein DM, Shu S, Howson R *et al.* (2012) Phytozome: a comparative platform for green plant genomics. *Nucleic Acids Res* **40**: D1178-D86.

Hebbali A (2020) olsrr: Tools for building OLS Regression Models. R package version 0.5.3. https://CRAN.R-project.org/package=olsrr

Katoh K, Standley DM (2013) MAFFT multiple sequence alignment software version 7: improvements in performance and usability. *Mol Biol Evol* **30**: 772-80.

Kiniry J, Johnson MV, Mitchell R *et al.* (2011) Switchgrass leaf area index and light extinction coefficients. *Agron J* **103**: 119-22.

Kiniry JR, Tischler CR, Van Esbroeck GA (1999) Radiation use efficiency and leaf CO_2 exchange for diverse C_4 grasses. *Biomass Bioenergy* **17**: 95-112.

Kõljalg U, Nilsson RH, Abarenkov K *et al.* (2013) Towards a unified paradigm for sequence-based identification of fungi. *Mol Ecol* **22**: 5271–7.

Lee MR, Hawkes CV (2021) Plant and soil drivers of whole-plant microbiomes: variation in switchgrass fungi from coastal to mountain sites. *Phytobioimes J* **5**: 69-79.

Li H, Durbin R (2010) Fast and accurate long-read alignment with Burrows-Wheeler transform. *Bioinformatics* **26**: 589-95.

Li H, Handsaker B, Wysoker A *et al.* (2009) The Sequence Alignment/Map format and SAMtools. *Bioinformatics* **25**: 2078-9.

Li Y, Hou R, Tao F (2021) Wheat morpho-physiological traits and radiation use efficiency under interactive effects of warming and tillage management. *Plant Cell Environ* **44**: 2386-401.

Lovell JT, MacQueen AH, Mamidi S *et al.* (2021) Genomic mechanisms of climate adaptation in polyploid bioenergy switchgrass. *Nature* **590**: 438-44.

Lozano YM, Aguilar-Trigueros CA, Roy J *et al.* (2021) Drought induces shifts in soil fungal communities that can be linked to root traits across 24 plant species. *New Phytologist* **232**: 1917-29.

Lundberg DS, Yourstone S, Mieczkowski P *et al.* (2013) Practical innovations for high-throughput amplicon sequencing. *Nat Methods* **10**: 999-1002.

Martin M (2011) Cutadapt removes adapter sequences from high-throughput sequencing reads. *EMBnet J* **17**: 10-2.
McMurdie PJ, Holmes S (2014) Waste not, want not: why rarefying microbiome data is inadmissible. *PLoS Computational Biology* **10**: e1003531.
Nguyen NH, Song Z, Bates ST *et al.* (2016) FUNGuild: An open annotation tool for parsing fungal community datasets by ecological guild. *Fungal Ecol* **20**: 241-6.
Nordberg H, Cantor M, Dusheyko S *et al.* (2014) The genome portal of the Department of Energy Joint Genome Institute: 2014 updates. *Nucleic Acids Res* **42**: D26-D31.
Oksanen J, Blanchet FG, Friendly M *et al.* (2022) vegan community ecology package version 2.6-2. https://cran.r-project.org/package=vegan
Pradhan S, Sehgal VK, Bandyopadhyay KK *et al.* (2018) Radiation interception, extinction coefficient and use efficiency of wheat crop at various irrigation and nitrogen levels in a semi-arid location. *Indian J Plant Physiol* **23**: 416-25.
Quinlan AR, Hall IM (2010) BEDTools: a flexible suite of utilities for comparing genomic features. *Bioinformatics* **26**: 841-2.
Smith DP, Peay KG (2014) Sequence depth, not PCR replication, improves ecological inference from next generation DNA sequencing. *PLoS One* **9**: e90234.
Wang Q, Garrity GM, Tiedje JM *et al.* (2007) Naive Bayesian classifier for rapid assignment of rRNA sequences into the new bacterial taxonomy. *Appl Env Micro* **73**: 5261-7.
Whitaker BK (2023) brikw/Whitaker_etal_CropFoliarFungiLandscape: updated release. Zenodo: v1.2.0, doi: 10.5281/zenodo.7888432.
Whitaker BK, Rua MA, Mitchell CE (2015) Viral pathogen production in a wild grass host driven by host growth and soil nitrogen. *New Phytologist* **207**: 760-8.
